# Algal Metabolites as Novel Therapeutics Against Methicillin-Resistant *Staphylococcus aureus* (MRSA): A Review

**DOI:** 10.3390/pharmaceutics17080989

**Published:** 2025-07-30

**Authors:** Ibraheem Borie M. Ibraheem, Reem Mohammed Alharbi, Neveen Abdel-Raouf, Nouf Mohammad Al-Enazi, Khawla Ibrahim Alsamhary, Hager Mohammed Ali

**Affiliations:** 1 Botany and Microbiology Department, Faculty of Science, Beni-Suef University, Beni-Suef 62521, Egypt; neveenabdelraouf@science.bsu.edu.eg (N.A.-R.); hagermohammed179@science.bsu.edu.eg (H.M.A.); 2 Biology Department, Science College, University of Hafr Al-Batin, Hafr Al-Batin 39524, Saudi Arabia; rmalharbi@uhb.edu.sa; 3 Department of Biology, College of Science and Humanities in Al-Kharj, Prince Sattam Bin Abdulaziz University, Al-Kharj 11942, Saudi Arabia; n.alenazi@psau.edu.sa (N.M.A.-E.); k.alsamhary@psau.edu.sa (K.I.A.)

**Keywords:** *Staphylococcus aureus*, MRSA, multidrug resistance, algal metabolites, bioactive compounds, antibacterial agents

## Abstract

Methicillin-resistant *Staphylococcus aureus* (MRSA), a multidrug-resistant pathogen, poses a significant threat to global healthcare. This review evaluates the potential of marine algal metabolites as novel antibacterial agents against MRSA. We explore the clinical importance of *S. aureus*, the emergence of MRSA as a “superbug”, and its resistance mechanisms, including target modification, drug inactivation, efflux pumps, biofilm formation, and quorum sensing. The limitations of conventional antibiotics (e.g., β-lactams, vancomycin, macrolides) are discussed, alongside the promise of algal-derived compounds such as fatty acids, pigments, polysaccharides, terpenoids, and phenolic compounds. These metabolites exhibit potent anti-MRSA activity by disrupting cell division (via FtsZ inhibition), destabilizing membranes, and inhibiting protein synthesis and metabolic pathways, effectively countering multiple resistance mechanisms. Leveraging advances in algal biotechnology, this review highlights the untapped potential of marine algae to drive innovative, sustainable therapeutic strategies against antibiotic resistance.

## 1. Introduction

Multidrug resistance (MDR) in bacterial pathogens, particularly methicillin-resistant *Staphylococcus aureus* (MRSA), represents a global health crisis, with the World Health Organization classifying MRSA as a “high-priority” pathogen due to its resistance to multiple antibiotics, including β-lactams, vancomycin, and macrolides [1,2]. Overuse of antibiotics in clinical and agricultural settings has accelerated resistance, leading to ineffective treatments, mortality rates exceeding 20% in invasive MRSA infections, and increased healthcare costs [3,4]. MRSA employs diverse resistance mechanisms, such as penicillin-binding protein (PBP2a) mutations, *mecA* gene acquisition, efflux pumps, quorum sensing, and biofilm formation, enabling survival under antibiotic pressure [5,6]. Resistance to last-resort drugs like vancomycin-resistant *S. aureus* (VRSA) and daptomycin underscores the urgent need for novel therapeutics [7]. Marine algae, adapted to extreme environments, produce secondary metabolites with potent antibacterial properties, including polyphenols (e.g., phlorotannins), terpenoids, fatty acids, and polysaccharides (e.g., ulvan, fucoidan), which target non-canonical pathways like bacterial membrane disruption and biofilm inhibition [8,9,10,11,12]. This review synthesizes the structural diversity, biosynthetic origins, and anti-MRSA mechanisms of algal metabolites, evaluating their therapeutic potential as sustainable solutions to combat MDR pathogens.

The urgency of this search is continually echoed in the most current scientific literature. A recent comprehensive review in 2024 by Khan et al. also highlighted the growing threat of MRSA and underscored the significant therapeutic potential of marine natural products as a primary source for new antimicrobial leads [13]. Building upon this established and timely relevance, the present review provides a unique and comprehensive contribution by explicitly bridging the fields of clinical microbiology and marine pharmacology. Its novelty lies in systematically mapping the complex, multifaceted resistance mechanisms of MRSA to the specific, multi-target modes of action of an extensive range of algal metabolite classes. By offering this integrated perspective, this paper serves as a one-stop resource that not only summarizes the state-of-the-art but also critically frames the potential of algal biotechnology as a sustainable solution to the urgent problem of antibiotic resistance, thereby guiding future research toward the most promising therapeutic avenues.

## 2. Search Strategy and Selection Criteria

A comprehensive literature search was conducted to identify relevant studies for this review. The search was performed using major scientific databases, including PubMed, Scopus, Google Scholar, and Web of Science. Keywords used in the search strategy included, but were not limited to “MRSA”, “methicillin-resistant *Staphylococcus aureus*”, “algae”, “marine algae”, “seaweed”, “cyanobacteria”, “antibacterial”, “anti-MRSA”, “bioactive compounds”, “phlorotannins”, “terpenes”, “fucoidan”, “alkaloids”, and “polysaccharides”.

The selection criteria for inclusion were as follows; articles published in English between January 2000 and May 2025 were considered. Only studies that evaluated the direct or indirect anti-MRSA activity of algal-derived compounds or extracts were included in the final review.

## 3. Detailed Study Related to *Staphylococcus aureus*

### 3.1. The Prevalent Traits of Staphylococcus aureus

*Staphylococcus aureus*, identified by Alexander Ogston in 1880, is a Gram-positive bacterium from the Micrococcaceae family, typically forming grape-like clusters [14]. It optimally grows at 37 °C and pH 7.4, tolerates high salt, and grows aerobically and anaerobically [15]. Characterized by a capsule, golden pigment, and mannitol fermentation, *S. aureus* forms distinctive colonies with hemolytic rings on blood agar. It is oxidase-negative, catalase-positive, and coagulase-positive, enabling plasma clotting for immune defense. Naturally found on human skin, in nasal passages, and mucous membranes without causing symptoms, *S. aureus* prefers the warm, moist nasal epithelium [16]. It spreads via aerosols and direct contact, posing significant risks in healthcare and community settings, causing infections from mild skin issues to severe diseases like bacteremia, endocarditis, osteomyelitis, and pneumonia [17].

### 3.2. Staphylococcus aureus as a Superbug and Emergence of MRSA

**Figure 1:**
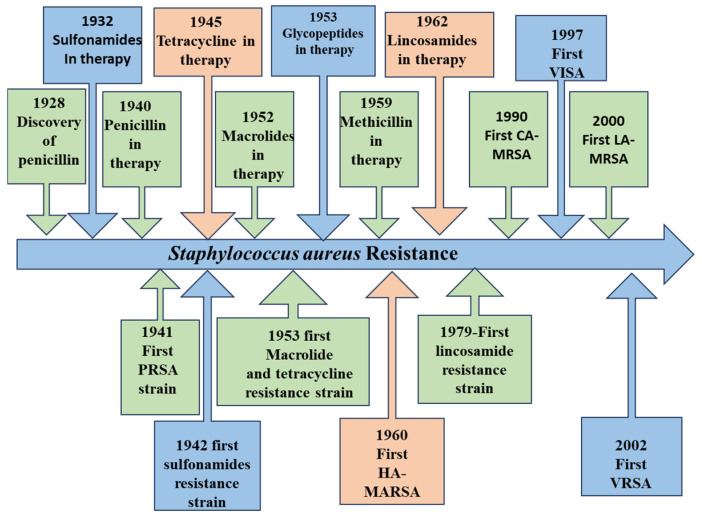
The evolution of *S. aureus* resistance over time [11].

*Staphylococcus aureus* rapidly developed antibiotic resistance (see Figure 1), becoming a dangerous pathogen due to antibiotic misuse in medicine and agriculture, heavy antibiotic usage, and the spread of antimicrobial resistance genes from increased anthropogenic activity. Penicillin, discovered in 1928 [18], was initially effective but faced resistance by 1945 [19] due to a plasmid-encoded enzyme degrading its β-lactam ring. This led to methicillin’s development in 1959 [20], a novel semisynthetic penicillin resistant to penicillinase. However, the methicillin-sensitive *S. aureus* chromosomal element (SCCmec) incorporated a gene (mecA) producing penicillin-binding protein 2a or 2′, leading to resistance, as reported by Jevons in 1961, just two years after methicillin’s introduction. PBP2a also allows bacterial multiplication in the presence of β-lactam inhibitors, resulting in MRSA emergence [21]. MRSA is a deadly multidrug-resistant “superbug” present in hospitals and communities worldwide, particularly in North Africa, East Asia, and Europe. It is linked to severe, high-fatality infections, significantly impacting immunocompromised patients. MRSA prevalence has risen globally, with risk factors including long hospital stays, open wounds, and catheter use. Treatment is challenging due to variable drug susceptibility in MRSA clones. Though vancomycin was a key treatment from 1958 [22], vancomycin-resistant strains (VRSA) were identified by 2002, further complicating treatment [23].

### 3.3. Mechanisms of Action of Current Antibiotics Against MRSA and Associated Challenges

**Figure 2:**
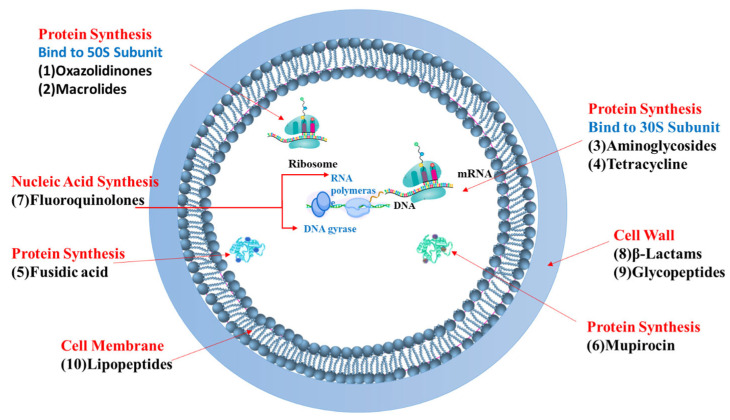
The fundamental targets of antibacterials against *S. aureus*. Legend: (1) Oxazolidinones block protein synthesis by binding to the ribosomal peptidyl transferase center of the 23S rRNA within the 50S ribosomal subunit. (2) Macrolides inhibit protein synthesis by interfering with the peptidyl transferase activity of the 50S ribosomal subunit. (3) Aminoglycosides inhibit protein synthesis by linking to the A-site (tRNA acceptor site) of the 16S rRNA within the 30S ribosomal subunit. (4) Tetracycline inhibits bacterial protein synthesis by binding to the 30S ribosomal subunit, blocking tRNA from binding at the acceptor site. (5) Fusidic acid inhibits bacterial protein synthesis and the translocation process by adhering to microbial elongation factor G (EF-G) and prevents the subsequent cycle of aa-tRNA attaching. (6) Mupirocin halts the bacterial protein synthesis by inhibiting the ileS gene encodes the enzyme isoleucyl-tRNA synthetase, blocking isoleucine from being incorporated into protein chains. (7) Fluoroquinolones target DNA topoisomerase and DNA gyrase enzymes, which facilitate DNA uncoiling during replication. (8) Penicillin inhibits bacterial cell wall synthesis by targeting penicillin-binding proteins. (9) Glycopeptides inhibit bacterial cell wall synthesis by targeting C-terminal D-Ala-D-Ala residue of the pentapeptide in the peptidoglycan precursor lipid II. (10) Lipopeptides rupture the cytoplasmic membrane, causing intrinsic ion leakage and the fast dying of cells to ensue by attaching to the plasma membrane in a calcium-dependent way.

**Figure 3:**
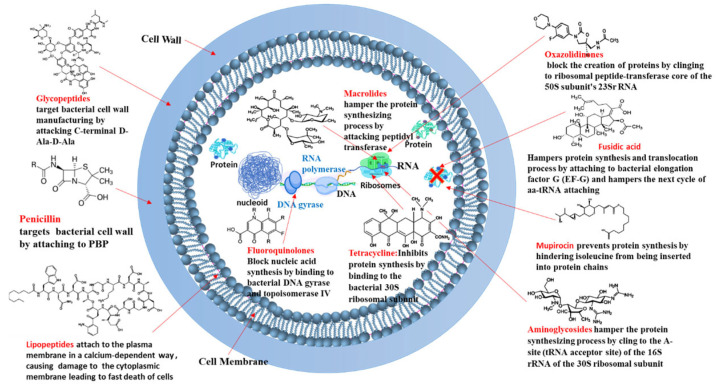
Chemical structure of various antibiotics and the primary molecular and structural targets of MRSA antibacterials.

**Figure 4:**
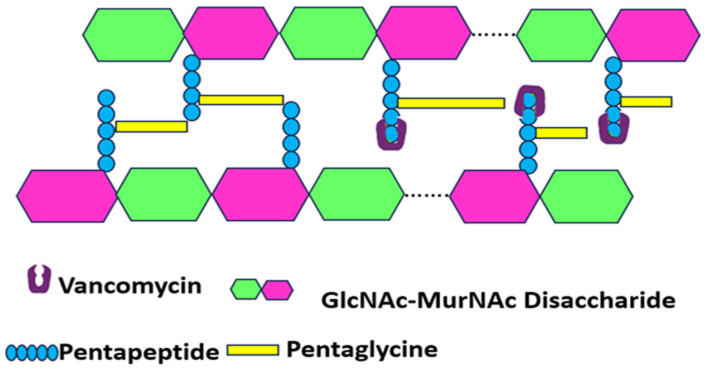
The antimicrobial activity of vancomycin versus *S. aureus*.

**Figure 5:**
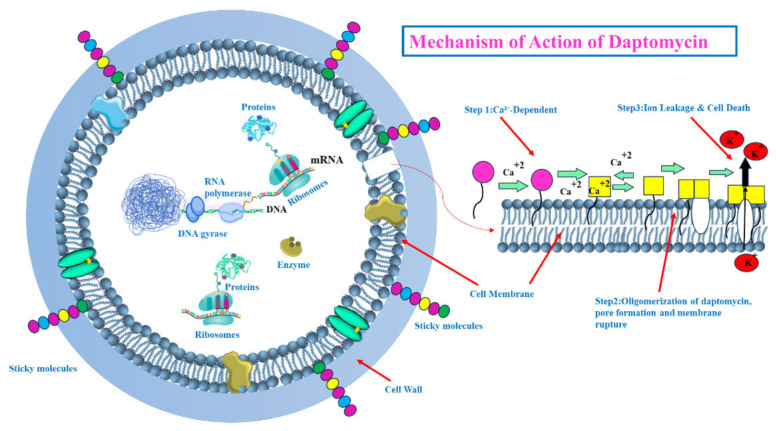
Daptomycin’s mode of action. Theoretical steps: (1) daptomycin attaches to the plasma membrane in a calcium-dependent way; (2) daptomycin assembles, rupturing the membrane; (3) intrinsic ion leakage and fast dying of cells ensue.

To combat *S. aureus* infections, including MRSA, various antibiotics have been developed that target different essential pathways within the bacterial cell (as illustrated in Figure 2 and Figure 3). The primary mechanisms of these antimicrobial agents can be broadly categorized as follows:

- Targeting Bacterial Cell Wall Synthesis: This is a common strategy. For instance, β-lactam antibiotics (e.g., penicillin and its derivatives, see Figure 3) disrupt peptidoglycan synthesis by inhibiting penicillin-binding proteins (PBPs), which are crucial for cross-linking peptidoglycan layers, leading to cell lysis [24,25]. Glycopeptides, such as vancomycin, bind directly to the D-alanyl-D-alanine termini of peptidoglycan precursors, thereby preventing transglycosylation and transpeptidation steps in cell wall assembly [21] (see Figure 4). Despite their initial effectiveness, the emergence of resistance, such as MRSA against methicillin and the rise in vancomycin-resistant strains (VRSA), limit their utility [26,27].

- Targeting the Bacterial Cell Membrane: Some antibiotics, like lipopeptides (e.g., daptomycin), compromise the integrity of the bacterial cell membrane. Daptomycin, in a calcium-dependent manner, inserts into the cell membrane, causing depolarization and pore formation, which leads to ion leakage and cell death [28,29] (see Figure 5). However, the efficacy of certain agents in this class can be affected by environmental factors, like daptomycin’s inactivation by pulmonary surfactants, restricting its use for specific infections [29].

- Inhibiting Bacterial Protein Synthesis: The bacterial ribosome (with its 30S and 50S subunits) is a critical target for many antibiotic classes (see Figure 3):
  ○ Antibiotics like tetracyclines and aminoglycosides target the 30S ribosomal subunit. Tetracyclines typically block the binding of aminoacyl-tRNA to the A-site of the ribosome [30,31,32,33,34], while aminoglycosides bind to the A-site causing misreading of mRNA and production of aberrant proteins [35,36].
  ○ Macrolides and oxazolidinones (e.g., linezolid) target the 50S ribosomal subunit. Macrolides often obstruct the peptide exit tunnel [37,38,39,40,41,42] and linezolid prevents the formation of the 70S initiation complex essential for starting protein translation [43,44,45,46].
  ○ Other antibiotics interfere with different components of the protein synthesis machinery; for example, fusidic acid inhibits elongation factor G (EF-G) [25] and mupirocin inhibits isoleucyl-tRNA synthetase [37], thereby halting protein production.
- Inhibiting Bacterial Nucleic Acid Replication: Fluoroquinolones interfere with DNA replication by inhibiting essential enzymes like DNA gyrase and topoisomerase IV (see Figure 3). These enzymes are vital for DNA uncoiling, replication, and segregation, and their inhibition leads to strand breaks and cell death [47,48,49,50,51].

Figure 2 and Figure 3 provide a general overview of these molecular targets and the chemical nature of various antibiotics. While these diverse mechanisms have been instrumental in treating bacterial infections, the remarkable ability of MRSA to develop resistance against many of these agents (as will be detailed in Section 3.4) underscores the urgent need for exploring and developing novel therapeutic approaches, such as those potentially offered by natural compounds from algae.

### 3.4. Staphylococcus aureus Resistance Mechanisms to Antimicrobial Agents

#### 3.4.1. Efflux Pumps

**Figure 6:**
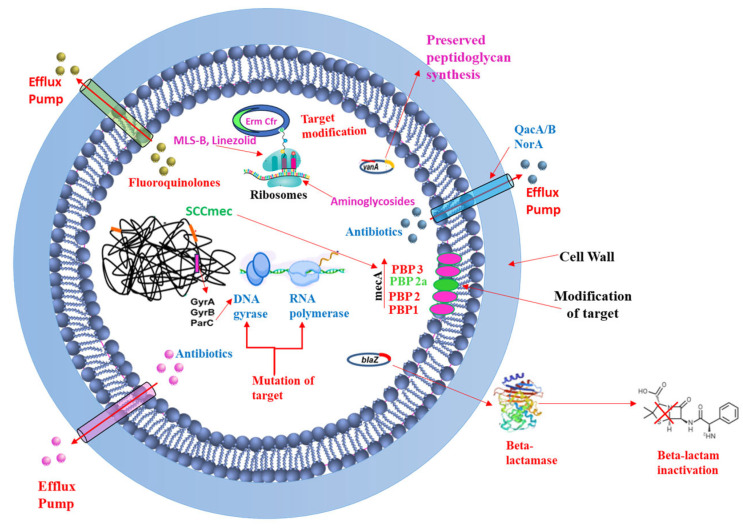
Summary of key resistance mechanisms in *Staphylococcus aureus*, including target site modifications, efflux pumps, acquisition of resistance genes and enzymatic deactivation, against major antibiotic classes like beta-lactams, glycopeptides, macrolide–lincosamide–streptogramin B antibiotics (MLSB), aminoglycosides, and fluoroquinolones.

Efflux pumps are protein structures in bacterial cell membranes that actively expel antibiotics and other substances, reducing drug efficacy and contributing to antibiotic resistance (see Figure 6). First found in *E. coli*, these pumps are present in most bacterial species, helping them survive in environments with drugs [52]. Efflux pumps are categorized into five major classes: Major Facilitator Superfamily (MFS), Resistance-Nodulation-Division (RND), Small Multidrug Resistance (SMR), ATP-Binding Cassette (ABC), and Multidrug and Toxic Compound Extrusion (MATE). Each type uses either proton gradients or ATP hydrolysis for energy [53]. They can expel a wide variety of drugs (MDR pumps) or specific ones [54]. In *Staphylococcus aureus*, pumps such as Quaternary ammonium compound resistance protein A (QacA) and Norfloxacin resistance protein A (NorA) are vital for resistance, especially in MRSA strains [55]. Beyond synthetic antibiotics, efflux pumps also assist in biofilm formation and protect against host-derived antimicrobials, making them a significant area of study in bacterial pathogenicity [56].

#### 3.4.2. Acquisition of Resistance Genes

Bacteria primarily acquire resistance genes through horizontal gene transfer (transformation, transposition, conjugation), gaining genetic material that confers resistance [57]. They can also undergo mutations in genes related to therapeutic targets, transport systems, or antibiotic-altering enzymes [58]. Plasmid-mediated gene transfer is the most common method (see Figure 6), spreading resistance genes between species like *Staphylococcus aureus* and *Enterococcus* [57]. While beneficial for bacteria, resistance mutations can slow their growth, as seen in methicillin-resistant *S. aureus* (MRSA) [37]. Using sub-inhibitory antibiotic concentrations fosters resistance by selecting hypermutable bacterial strains and increasing the transfer of mobile genetic elements [59].

#### 3.4.3. Target Modification

Bacteria resist antibiotics by modifying target sites to reduce drug binding [60]. This common mechanism involves enzymatic changes, gene alterations, or replacing the original target. For instance, *S. aureus* alters penicillin-binding proteins (PBPs) to lessen β-lactam binding and modifies DNA gyrase to resist fluoroquinolones (see Figure 6) [61]. Resistance to ribosome-targeting drugs occurs when the erm gene methylates ribosomal subunits, blocking macrolide binding. Mutations in RNA polymerase provide rifampicin resistance [62], while changes in cell membrane charge via mprF mutations prevent daptomycin binding [63]. Additionally, van genes modify cell wall structure, decreasing vancomycin’s effectiveness (see Figure 6) [64].

#### 3.4.4. Drug Inactivation

Bacteria inactivate antibiotics by breaking down or modifying the drug’s active part. Key methods include producing enzymes like β-lactamase, which destroys penicillin’s β-lactam ring (see Figure 6) [65]. Other bacteria modify drugs such as aminoglycosides and chloramphenicol through acetylation, phosphorylation, or adenylation, blocking their action [66]. For example, *S. aureus*, *Streptococcus pneumoniae*, and *Enterococcus faecalis* make enzymes that alter aminoglycosides, reducing their binding to bacterial ribosomes [67]. Furthermore, the tetX gene can inactivate tetracycline by hydrolysis, and chloramphenicol acetyltransferase prevents chloramphenicol binding by acetylating it [38].

#### 3.4.5. Persister Cells

**Figure 7:**
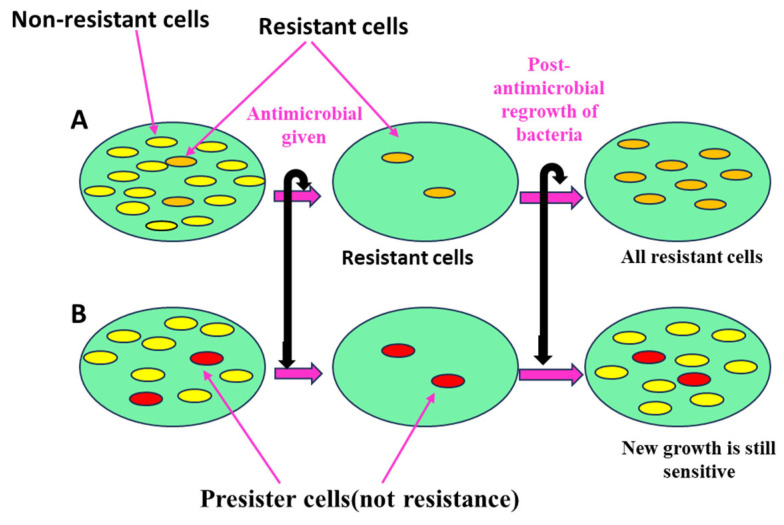
Persistence versus resistance: There are two probable results when antimicrobial medications are introduced to bacterial cells. It is possible that certain cells possess the capability to resist antibacterial drugs (**A**). Just the resistant cells remain after the non-resistant ones are eliminated. The whole of the cells in the culture will be able to resist the antibacterial medications upon the regrowth of the resistant cells. The alternative explanation is the presence of persister cells, which are inactive rather than resistant (**B**). Only the persister cells remain after the non-persister cells are eliminated. The cells that are not in a state of dormancy will still be vulnerable to the antibacterial drug when the persister cells proliferate again [57].

Persister cells are dormant, slow-growing bacteria that tolerate antibiotics without genetic resistance [68]. These phenotypic variants survive high antibiotic doses through reduced metabolic activity, particularly in biofilms (see Figure 7), and constitute about 1% of stationary-phase cultures [69]. They evade antibiotics by lowering energy use, using toxin–antitoxin systems, repairing DNA, and decreasing ATP levels, which reduces antibiotic target activity [68]. Persisters can resume growth after antibiotic exposure ends, contributing to persistent infections and making complete bacterial eradication difficult. This tolerance, sometimes associated with programmed cell death mechanisms, poses challenges for effective infection control [70].

#### 3.4.6. Quorum Sensing

Quorum sensing is a bacterial communication process used to regulate group behaviors like biofilm formation, virulence, and resistance [71]. Bacteria achieve this by detecting signal molecules known as autoinducers [72]. When these autoinducers reach a certain threshold level corresponding to the bacterial population size, specific genes are activated to coordinate collective actions [73].

**Figure 8:**
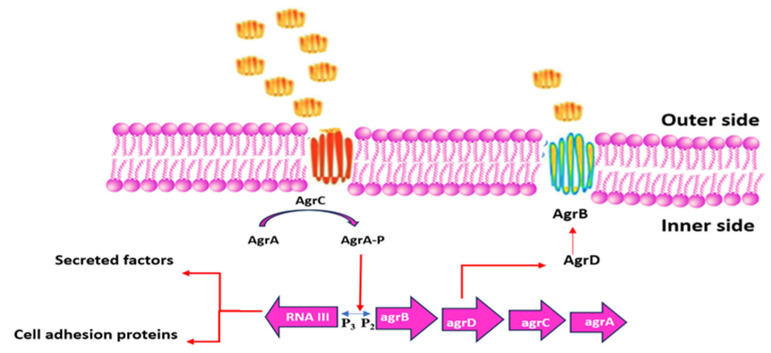
*Staphylococcus aureus*’s quorum sensing.

In *Staphylococcus aureus*, quorum sensing is directed by autoinducing peptides [50] and involves the accessory gene regulator (Agr) system. This system influences factors such as toxin secretion and biofilm development (see Figure 8) [74]. Quorum sensing allows bacteria to adapt, survive, and spread. Consequently, inhibiting quorum sensing can reduce bacterial virulence and biofilm formation, potentially improving infection control strategies [75].

#### 3.4.7. Biofilm-Mediated Resistance

Biofilms are protective, complex microbial communities adhered to surfaces, producing an extracellular polymeric matrix of DNA, proteins, and polysaccharides [76]. This matrix strengthens bacterial defense against antibiotics and immune responses, contributing to biofilm-mediated resistance [77]. Quorum sensing regulates gene expression in biofilms, coordinating growth and detachment. Bacteria within biofilms exhibit significantly higher antibiotic resistance, up to 1000 times greater than free-floating (planktonic) cells [78]. In *Staphylococcus aureus*, the methicillin-resistant gene A (mecA gene) enhances biofilm formation by suppressing the accessory gene regulator (agr) system, which normally limits biofilm growth. The biofilm matrix restricts antibiotic penetration and facilitates horizontal gene transfer of resistance. Consequently, biofilms enable bacteria to enter a low-metabolic, persistent state, shielded from antibiotics, allowing survival and reinfection after antibiotic removal [79]. Together, Figure 6 and Figure 7 highlight the complexity of combating *S. aureus*, particularly MRSA, and emphasize the need for continuous research to overcome emerging resistance mechanisms, providing a foundation for designing next-generation antibiotics and therapeutic approaches.

## 4. Algal Metabolites with Anti-MRSA Activity

The growing crisis of antibiotic resistance, particularly with methicillin-resistant *Staphylococcus aureus* (MRSA), necessitates the urgent development of novel antimicrobial agents. Marine algae, a vast and diverse group of photosynthetic organisms, are a rich source of bioactive secondary metabolites with promising antimicrobial properties. These metabolites offer diverse chemical structures and mechanisms of action, presenting opportunities to overcome MRSA’s resistance mechanisms. 

This comprehensive document details 24 algal metabolites related to more than 10 chemical groups with potential anti-MRSA activity, outlining their chemical composition, algal sources, mechanisms of action, and considerations for their development, as summarized in Table 1.

Important Considerations Before Diving In

- Specificity: While many of these compounds show promising in vitro activity against MRSA, it is crucial to remember that their efficacy and safety in vivo (animal or human) models need to be thoroughly investigated. In vitro activity does not always translate to clinical effectiveness.
- Mechanism of Action: The mechanisms described are based on current scientific understanding and may be incomplete or subject to revision as more research is conducted.
- Extraction and Purification: Obtaining pure compounds from algae can be challenging and costly. This is a significant factor in developing algal metabolites into viable therapeutics.
- Bioavailability: The ability of these compounds to be absorbed, distributed, metabolized, and excreted (ADME) in a living organism is crucial for their therapeutic potential. This needs to be studied extensively.

**Figure 9:**
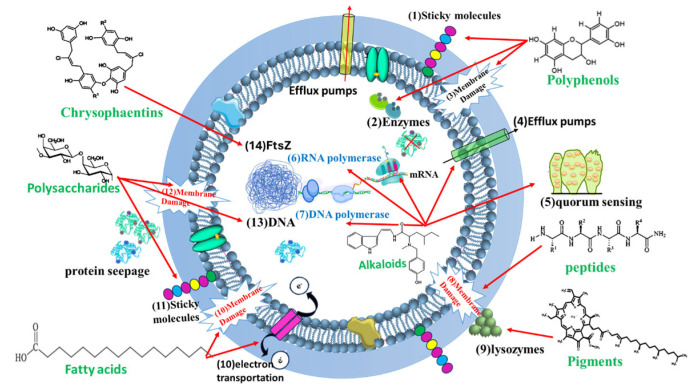
Illustrates the antibacterial mechanisms of algal metabolites against methicillin-resistant *Staphylococcus aureus* (MRSA).

Algal metabolites have emerged as promising antibacterial agents due to their ability to target multiple critical sites within bacterial cells. As illustrated in Figure 9, these bioactive compounds exert their effects by disrupting bacterial cell walls, cell membranes, DNA replication and integrity, and essential metabolic pathways. The diverse mechanisms of action demonstrated by algal metabolites highlight their potential as effective alternatives or complements to conventional antibiotics, particularly in the face of rising antimicrobial resistance. This multi-target approach not only enhances their antibacterial efficacy but also reduces the likelihood of resistance development [96].

### 4.1. Polyphenols

Polyphenols are water-soluble phenolic compounds found abundantly in the vacuoles of both terrestrial and marine species, with algae as a primary source of known polyphenols. These compounds have a diverse chemical structure, commonly consisting of benzene rings bonded with multiple hydroxy groups [168]. Over 8000 distinct phenolic structures are recognized, including simple phenolic acids like hydroxybenzoic acid and complex compounds such as bromophenols and phlorotannins [169]. The concentration of polyphenols in algae varies depending on factors such as environment, season, and extraction method, but these secondary metabolites do not influence core biological processes like photosynthesis or reproduction [170].

**Figure 10:**
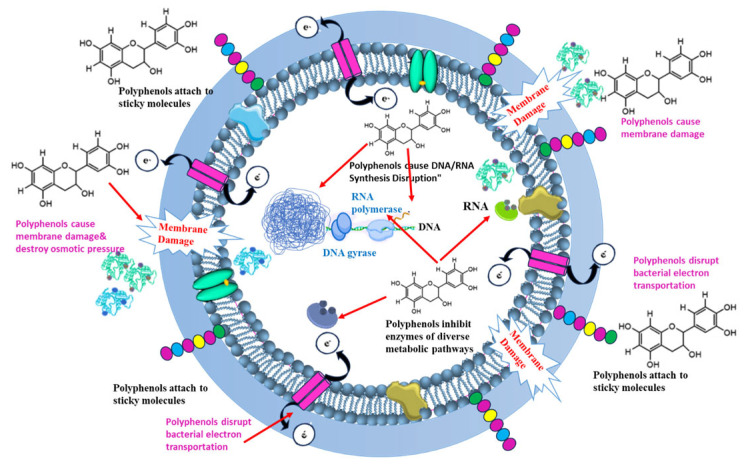
A graphic illustrating the various ways in which polyphenols exhibit antibacterial properties.

Polyphenols in algae are typically released in response to environmental stressors, offering protection through their antioxidant properties that help alleviate cellular oxidative stress [171]. The antioxidative potential is attributed to the presence of phenolic rings, which can stabilize oxidizing radicals by capturing electrons [172]. Polyphenols derived from algae display a wide range of biological activities, including anti-inflammatory, antibacterial, antifungal, anticancer, and antidiabetic effects [173]. The antimicrobial action of polyphenols varies with polymerization and hydroxylation levels, targeting various cellular sites, including attaching to sticky molecules on the surface, cell walls, rupturing the membrane permeability, and inhibiting enzymes of diverse metabolic pathways (see Figure 9 and Figure 10) [96].

Studies show that compounds such as phlorotannin, fucoxanthin, and fecosterol are isolated from *Padina australis* and inhibit bacterial growth, including that of methicillin-resistant *Staphylococcus aureus* (MRSA) [80]. Phlorofucofuroeckol from some brown macroalga exhibits potent antibacterial properties against MRSA by damaging the cell membrane and inhibiting methicillin-resistance genes [81]. Furthermore, extracts from *Padina antillarum* and *P. boergeseni* show antibacterial effects against *Staphylococcus aureus* and *Bacillus subtilis* but not against Gram-negative bacteria like *Klebsiella pneumoniae* or *Pseudomonas aeruginosa* [174]. Crossbyanol B, derived from *Leptolyngbya crossbyana*, is particularly effective against MRSA and in brine shrimp assays, attributed to its sulfa substituents [90]. Algal cells accumulate substantial quantities of polyphenols such as phlorotannins, bromophenols, phenolic acids, and flavonoids, with red and green algae containing especially high levels of these compounds [175].

#### 4.1.1. Phlorotannins

Phlorotannins are a class of polyphenols primarily found in brown algae, with lesser amounts in red and green algae [176]. *Ecklonia kurome, Laminaria digitata, Fucus vesiculosus*, and *Sargassum* sp. are all examples of brown algae species known to produce phlorotannins. These compounds are polymers of phloroglucinol (1,3,5-trihydroxybenzene) and are categorized based on their bonding types, such as fucophlorethols, carmalols, and phlorethols [177]. Some brown algae also contain halogenated forms of phlorotannins, which can make up as much as 20% of the algae’s dry mass [178]. Phlorotannins, varying in molecular weight from 126 Da to 650 kDa, are synthesized via the acetate–malonate pathway within the Golgi apparatus [179]. These polyphenols accumulate in specific granules called physodes, comprising up to 90% of the total phlorotannin content. Additionally, algae can contain sulfated or halogenated forms of phlorotannins, which exhibit strong antioxidant properties by scavenging free radicals [180]. The bactericidal effectiveness of phlorotannins is influenced by their polymerization degree, impacting both aerobic and anaerobic bacteria differently based on cell membrane structures [82].

**Mechanism of Action Against MRSA**: Phlorotannins exhibit a multifaceted approach to inhibiting MRSA including the following:

**a.**  **Biofilm Inhibition**: Phlorotannins interfere with the formation and maturation of MRSA biofilms, reducing bacterial adherence and virulence.
**b.**  **Quorum Sensing Interference**: Phlorotannins can disrupt quorum sensing systems, which regulate gene expression and virulence in MRSA [83].
**c.**  **Cell Membrane Disruption**: Phlorotannins’ lipophilic properties enhance their interaction with bacterial cell membranes, leading to irreversible damage, inhibiting oxidative phosphorylation [96], cellular component coagulation, increasing its permeability and leading to cell lysis [84].
**d.**  **Enzyme Inhibition:** Phlorotannins can chelate metal ions, such as iron and zinc, that are essential for the activity of bacterial enzymes involved in metabolism and virulence. By binding these metal ions, phlorotannins effectively inhibit these enzymes, disrupting vital pathways [96]. Furthermore, they affect antioxidant enzyme activity [85].
**e.**  **Nucleic acid disruption**: Phlorotannins interact with bacterial RNA and DNA, hindering cell replication and decreasing bacterial cell proliferation [86]. Additionally, phlorotannins can reduce methicillin resistance gene expression in MRSA, impeding methicillin resistance [87].

Nagayama et al. [88] demonstrated the potent antimicrobial activity of phlorotannins isolated from *Ecklonia kurome* against *Staphylococcus aureus*, including MRSA strains, highlighting their potential as therapeutic agents. Phlorotannins from *Ecklonia stolonifera*, have demonstrated bactericidal activity against a range of pathogens, including MRSA and *Campylobacter* sp. [82].

*Eisenia bicyclis* algae-derived phlorotannins, for example, exhibit antioxidant activity far greater than ascorbic acid, with MIC values as low as 32–64 μg/mL against MRSA and other bacteria [96]. Another species, *Ceratodictyon spongiosum*, yielded a phlorotannin with strong anti-MRSA properties, showing high antibacterial efficacy. Additionally, cladophorols from green algae like *Chaetoceros socialis* have potent antimicrobial properties, with certain compounds effective against MRSA at low MICs [89].

#### 4.1.2. Bromophenols

Bromophenols are halogenated organic compounds with bromine atoms bonded to a phenol backbone. The number and position of bromine atoms, as well as the presence of other substituents, contribute to the diversity of bromophenols. First identified in the algae *Neorhodomela larix*, these compounds are now known to be widespread across macroalgal taxa [91]. *Rhodomela larix*, *Laurencia obtusa*, and *Polysiphonia lanosa* are among the red algae (Rhodophyta) species known to produce bromophenols.

Bromination, a common reaction in marine environments, is facilitated by bromoperoxidase enzymes found in marine algae [96]. Bromophenols have ecological roles as defensive agents and possess biological activities, including anti-inflammatory, anti-tumor, and antidiabetic, antibacterial capabilities [92].

**Mechanism of Action Against MRSA**: Bromophenols target MRSA through multiple mechanisms:

**a.**  **Cell Membrane Disruption**: Like phlorotannins, bromophenols can insert into the bacterial cell membrane, disrupting its integrity and leading to cell leakage.
**b.**  **Quorum Sensing Inhibition:** Bromophenols are known quorum sensing inhibitors, interfering with the bacterial communication system that controls the expression of virulence factors such as biofilm formation and toxin production. By inhibiting quorum sensing, bromophenols reduce the ability of MRSA to cause infection.
**c.**  **Protein Inhibition:** Bromophenols have been shown to inhibit bacterial proteins essential for survival and replication.

Ganesan et al. [93] investigated bromophenols from red marine algae and found that they exhibited significant antioxidant and antibacterial activities, indicating their potential as therapeutic agents against MRSA and other pathogens.

Bromophenols derived from *Rhodomela confervoides* have demonstrated antibacterial action against multiple harmful strains, such as *P. aeruginosa* and *S. aureus* [94]. Additionally, bromophenols from *Kappaphycus* sp. showed activity against *S. aureus*, *Vibrio cholerae*, and *Pseudomonas fluorescens* [95].

Recent research continues to affirm the therapeutic potential of this class, not only through direct action but also through synergistic interactions with conventional antibiotics. For example, a 2024 study led to the isolation of a new bromophenol from the brown alga *Leathesia marina*. This novel compound not only exhibited direct anti-MRSA activity but, more importantly, demonstrated a significant synergistic effect when combined with oxacillin, substantially lowering the concentration of the antibiotic required to inhibit MRSA. This highlights a critical strategy where algal metabolites can be used to restore the efficacy of existing antibiotic classes facing resistance [181].

### 4.2. Alkaloids

The first discovery of alkaloids in the 18th century, with morphine from *Papaver somniferum* and hordenine from the alga *Phyllophora nervosa*, highlights their role as metabolic byproducts used in defense and growth regulation in algae. Alkaloids are often nitrogen-based compounds derived from amino acids, and though they typically exhibit basic properties, a few are mildly acidic or neutral due to elements like phosphorus and sulfur [97]. Alkaloids are primarily thought of as metabolic pathway byproducts. These substances function as defense agents against a range of predators and correspond to growth regulators in algae.

#### 4.2.1. Cyanobacterial Alkaloids

While cyanobacteria are better known for producing peptides (like microcystins and cyanopeptolins, which we described earlier), they can also produce countless types of alkaloids structural diversity including (Indoles, Pyrrolidines and Pyrrolizidines, Quinolines, Pyridines) which can be obtained from various cyanobacterial species, including (but not limited to) *Lyngbya* sp., *Nostoc* sp., and *Hapalosiphon* sp. However, the research on cyanobacterial alkaloids with specific anti-MRSA activity is less extensive than for other compound classes from cyanobacteria. Many cyanobacterial alkaloids are still under investigation.

**Prevalent Mechanisms of Action Against MRSA:** The mechanisms of action of cyanobacterial alkaloids against MRSA vary depending on the specific compound:

**a.**  **Interference with Quorum Sensing:** Some alkaloids can interfere with quorum sensing, a cell-to-cell communication system that MRSA uses to coordinate biofilm formation and virulence factor production. Berberine from cyanobacteria has been shown to inhibit gene regulation involved in biofilm formation [96].
**b.**  **Disruption of Cell Membranes:** Certain alkaloids may disrupt the integrity of bacterial cell membranes, leading to increased permeability and cell death. Alkaloids obtained from cyanobacteria possess the potential to suppress MRSA’s efflux pumps [98].
**c.**  **Inhibition of Bacterial Enzymes:** Some alkaloids may inhibit bacterial enzymes involved in cell wall synthesis, DNA replication, or protein synthesis.
**d.**  **DNA intercalation:** Some alkaloids can bind to and interfere with DNA production. Cyanobacterial alkaloids are capable of disrupting both MRSA’s transcription and translation processes by suppressing both DNA and RNA polymerases [98].

Mo et al. reported that the Hapalindole group of alkaloids, which are efficient against *Streptococcus* and *Staphylococcus* species, were produced from cyanobacteria such as *Hapalosiphon* fontinalis and *Fischerella* sp. [99]. Alkaloid-rich cyanobacteria such as *Fischerella* sp. demonstrate efficacy against a variety of bacterial species, while other cyanobacteria, like *Nostoc insulare*, produce norharmane alkaloids with broad antimicrobial activity against *Staphylococcus aureus* and *Vibrio* species [100].

At 150 μg/mL of alkaloid concentration, the first-ever isolated alkaloid compound N-methylcytisin from the cyanobacterium *Hapalosiphon* aureus was demonstrated to display potent antimicrobial action towards *S. aureus* [100]. Specific groups, such as Hapalindole alkaloids, are effective against *Streptococcus* and *Staphylococcus* species, with *Hapalosiphon aureus* producing N-methylcytisin, which exhibits significant antimicrobial activity without cytotoxic effects at tested doses [101]. Calothrixin A, from *Calothrix* sp., is another notable alkaloid with activity against *Bacillus* and *Staphylococcus species*. Further research on algal-derived alkaloids is needed to explore their antimicrobial mechanisms and potential therapeutic applications [96].

#### 4.2.2. Other Marine Algal Alkaloids

Algal alkaloids are predominantly sourced from marine species, including phenylethylamine, halogenated, and indole-based alkaloids, which exhibit unique halogenation specific to marine environments. Marine-derived alkaloid, such as caulerpin from *Caulerpa* species, particularly *Caulerpa taxifolia* (invasive green alga), has demonstrated strong antibacterial effects, particularly against resistant strains like MRSA [97,102].

Caulerpin is a red pigment and bisindole alkaloid (C_24_H_18_N_2_O_4_). Structurally, it consists of two indole rings linked together. The red color is due to the conjugated system of double bonds within the molecule.

**Mechanism of Action of Caulerpin Against MRSA:**

**a.**  **Disruption of Cell Membrane Function:** Caulerpin is believed to primarily act by disrupting the cell membrane function of bacteria, including MRSA. This disruption can lead to increased membrane permeability, leakage of cellular contents, and, ultimately, cell death. The lipophilic nature of caulerpin allows it to easily insert into the lipid bilayer of bacterial membranes.
**b.**  **Inhibition of Cell Division:** Caulerpin may also inhibit bacterial cell division, preventing MRSA from replicating.

The alkaloid contained in algae with green color (*Codium tomentosum*, *Ulva lactuca*), algae with brown color (*Dictyopteris membranacea*, *Sargassum vulgare*, and *Cystoseira barbata*), and algae with red color (*Gelidium latifolium*) differentially inhibited bacterial species that are Gram-positive (*Staphylococcus aureus*, *Staphylococcus epidermidis*, and *Bacillus subtilis*) as well as Gram-negative ones (*Pseudomonas aeruginosa*, *Salmonella typhimurium*, *Klebsiella pneumoniae*, and *E.coli*) [182].

In marine sponges, alkaloids like arenosclerins and haliclonacyclamine exhibit selective antibacterial activity, especially against strains like *S. aureus* and *P. aeruginosa*. These compounds did not affect *E. coli* or *Candida albicans*, underscoring their selective activity [183]. Other marine alkaloids, such as nakijinamines A from sponges, also display antibacterial impact against *S. aureus*, *Micrococcus luteus*, and *B. subtilis* [184].

Different algae species display varying antibacterial potency against both Gram-positive and Gram-negative bacteria, suggesting that environmental factors influence these bioactive compounds [103].

### 4.3. Pigments

Pigments are colorful compounds in algae that reflect visible wavelengths. Macroscopic algae are classified into three main groups—brown (Ochrophyta), red (Rhodophyta), and green (Chlorophyta)—based on their pigment content. Brown algae owe their color primarily to fucoxanthin, a dominant xanthophyll, while red algae have phycoerythrin-rich phycobiliproteins (PBPs) that give them their distinctive color. In contrast, the green color of green algae results from chlorophyll *a* and *b* [104].

Pigments are structurally categorized into open tetrapyrroles (PBPs), porphyrins, closed tetrapyrroles (e.g., chlorophyll *a* and *b*), and polyisoprenoids (carotenoids) [105]. This classification emphasizes three major pigment types: PBPs, carotenoids, and chlorophylls [104]. Pigments play key roles in photosynthesis and impart characteristic colors and photoprotective qualities to algae [96].

Due to their biological properties, pigments have significant economic interest. Studies indicate that pigments exhibit diverse bioactivities such as antibacterial, anticancer, antioxidant, antiangiogenic, anti-inflammatory, and anti-obesity effects, with applications in photodynamic therapy, drug delivery, photothermal therapy, and wound healing [106].

**Mechanism of Action Against MRSA:** Algal pigments can combat MRSA through multiple mechanisms:

**a.**  **Oxidative Stress Induction:** Pigments can generate reactive oxygen species (ROS), such as superoxide radicals and hydroxyl radicals, which damage bacterial cells. Sachindra et al. [107] demonstrated the antioxidant properties of fucoxanthin-rich extracts from *Laminaria japonica* and *Undaria pinnatifida* and highlighted their potential antibacterial activities.
**b.**  **Cell Membrane Disruption:** Some pigments can insert into the bacterial cell membrane, disrupting its structure and function.
**c.**  **Photosensitization:** When exposed to light, some pigments can generate singlet oxygen, a highly reactive form of oxygen that damages bacterial cells.

All algae contain chlorophylls—naturally green, lipid-soluble pigments with a porphyrin ring structure. Chlorophyll *a* is predominant in macroalgae, while chlorophylls *b* and *c* are also common, and red algae contain chlorophyll *d* [108]. Chlorophyll and its derivatives show antibacterial effects, with chlorophyll a and b effectively inhibiting bacteria like *Streptococci*, *Lactobacilli*, and *Staphylococci*, as well as oral pathogens *Fusobacterium nucleatum* and *Porphyromonas gingivalis* [109]. Chloroform extracts of *Caulerpa racemosa* containing phenolphthalein show antibacterial effects against *Escherichia coli* and MRSA [103]. Additionally, derivatives of chlorophyll *a* found in *Isochrysis galbana* exhibit antibacterial activity against opportunistic pathogens like *Micrococcus* and *Staphylococcus aureus* [110].

Additional pigments, like water-soluble phycobiliproteins, contribute fluorescent colors, with variants such as blue phycocyanins, red phycoerythrins, and light-blue allophycocyanins, particularly in cyanobacteria and red macroalgae [111]. Phycocyanin is a blue-pigmented protein complex (phycobiliprotein). It consists of apoprotein subunits (alpha and beta) covalently linked to phycocyanobilin, a linear tetrapyrrole chromophore that absorbs light. The chromophore gives phycocyanin its blue color. *Spirulina* (*Arthrospira platensis*) and *Aphanizomenon flosaquae* (both are cyanobacteria) are full of phycocyanin pigments.

**Mechanism of Action of Phycocyanin Against MRSA:** Phycocyanin possesses antioxidant, anti-inflammatory, and immunomodulatory activities. It can help reduce inflammation and protect tissues damaged by MRSA toxins. It has also demonstrated direct antibacterial effects by interfering with bacterial cell wall synthesis and disrupting membrane integrity. Additionally, phycocyanin can inhibit biofilm formation in MRSA and reduce the expression of virulence factors [112].

Carotenoids are lipid-soluble pigments that provide yellow, orange, and red colors and they are divided into xanthophylls and carotenes [108]. Carotenoids are the pigments that have been investigated the most antibacterial capabilities against MRSA; they are thought to work by accumulating lysozyme, an enzyme that breaks down bacterial cell walls [113].

Astaxanthin is a red–orange carotenoid pigment, a tetraterpenoid. Chemically, it is 3,3′-dihydroxy-β,β′-carotene-4,4′-dione (C_40_H_52_O_4_). It contains hydroxyl and keto groups, giving it strong antioxidant properties. *Haematococcus pluvialis* (green algae) is one of the main sources of astaxanthin.

**Mechanism of Action of Astaxanthin Against MRSA:** Astaxanthin’s primary mechanism is not direct bacterial killing but rather the reduction in oxidative stress caused by MRSA infection. MRSA infection triggers the production of reactive oxygen species (ROS) by immune cells. Astaxanthin, being a potent antioxidant, scavenges these ROS, protecting host tissues from damage. In addition, studies have shown that astaxanthin can directly inhibit biofilm formation in MRSA by interfering with quorum sensing and adhesion [114]. It is also believed that astaxanthin disrupts cell wall synthesis and bacterial cell membranes.

Carotenoids, especially fucoxanthin, demonstrate activity against both Gram-positive and Gram-negative bacteria, although Gram-positive bacteria are more strongly inhibited. Fucoxanthin’s antibacterial mechanisms are believed to involve cytoplasmic leakage, increased membrane permeability, and nucleic acid synthesis inhibition. Studies indicate that fucoxanthin is particularly effective against Gram-positive bacteria like *S. aureus*, *S. epidermidis*, and *S. agalactiae*, but not against certain Gram-negative bacteria like *K. pneumoniae* [115]. Extracts from algae such as *Laurencia obtusa* and *Ulva lactuca* have demonstrated significant antibacterial zones against pathogens like *S. aureus*, *Bacillus* subtilis, and *Pseudomonas aeruginosa* [116].

β-Carotene is a red–orange carotenoid pigment, a tetraterpenoid (C_40_H_56_). It is a pre-cursor to vitamin A. *Dunaliella salina* (green algae) and *Spirulina* sp. (*Arthrospira platensis*; cyanobacteria) are considered the main algal sources of β-Carotene.

**Mechanism of Action of β-Carotene Against MRSA:** Like astaxanthin, β-carotene acts as an antioxidant, reducing oxidative stress associated with MRSA infection. It can enhance the activity of immune cells like macrophages, improving their ability to phagocytose and kill MRSA. It may also have some direct antibacterial effects by disrupting the bacterial membrane, but these are generally weaker compared to other compounds [117]. These findings underscore the vast antimicrobial potential of algal pigments against MRSA, influenced by the algae’s source and environment [96].

### 4.4. Polysaccharides

Polysaccharides are complex biological macromolecules made of sugar monomers linked by glycosidic bonds. Their structural diversity arises from various sugars and derivatives, such as uronic acid, which can form multiple bonding configurations. They exhibit both linear and branched forms and may be neutral or acidic. These polysaccharides provide structural integrity to algal cell walls, contributing rigidity and strength. Algal cell walls feature complex structures, distinct from plant cell walls, with unique fiber architectures, and continuous exploration of marine polysaccharides for extraction holds promise [133]. Seaweed-derived polysaccharides exhibit antibacterial properties influenced by factors like molecular weight, sulfate content, and structural features. Their applications span medicine and food, utilizing their ability to interact with bacterial cell walls and membranes to disrupt cellular functions [185].

Polysaccharides display their antibacterial properties against MRSA by having the potential to link with bacterial cell wall-attached substances, plasma membranes, and DNA, leading to protein seepage, bacterial DNA attachment, and elevated cytoplasmic membrane permeability. Polysaccharides from *Pterocladia capillacea* and *Dictyopteris membranacea*, extracted using hot and cold water, inhibit growth in several bacteria, including *Staphylococcus aureus* and *Escherichia coli* [186].

Sulfated polysaccharides (SPs) from brown and red algae represent a promising class of natural compounds for combating MRSA infections, offering a multifaceted approach through adhesion inhibition, biofilm disruption, and immune modulation. In green, red, and brown algae, sulfated polysaccharides range widely from 4% to 76% of dry weight (DW). Green algae, for example, contain ulvan, a sulfated polysaccharide, while red algae primarily contain sulfated galactans, carrageenans, and agars. The primary polysaccharides in red macroalgae, galactans, consist of alternating β-D-galactopyranosyl and α-galactopyranosyl units [187]. Brown algae are notable for high concentrations of alginate and other polysaccharides such as laminarans and cellulose, essential for the structural composition of their cell walls [188].

#### 4.4.1. Ulvan

Ulvan is a complex sulfated polysaccharide found in the cell walls of green algae be-longing to the Ulva and Enteromorpha genera. It is a complex heteropolysaccharide composed of a variety of sugar residues and sulfate groups. It is primarily composed of rhamnose, xylose, glucuronic acid, and iduronic acid. The backbone structure is complex and irregular, with varying proportions of the different sugar monomers. The main repeating disaccharide units are ulvanobiuronic acid A3S and ulvanobiuronic acid B3S. It may contain smaller amounts of glucose, galactose, and mannose. It contains sulfate groups (SO_3_^−^) attached to the sugar residues. The degree and position of sulfation are highly variable and depend on the algal species, environmental conditions, and extraction methods. Its molecular weight varies depending on the source and extraction method, typically ranging from 20 kDa to 2000 kDa. *Ulva lactuca* (Sea Lettuce), *Ulva compressa, Ulva intesti-nalis*, and *Enteromorpha prolifera* (often now classified as *Ulva prolifera*) are the main algal sources for ulvan extraction.

**Mechanism of Action Against MRSA:** While direct studies on ulvan’s efficacy against MRSA are limited, insights from related Gram-positive bacteria, such as *Listeria monocytogenes*, suggest plausible mechanisms. For example, ulvan reduces bacterial attachment to epithelial cells [118], implying a role in blocking initial colonization. Additionally, ulvan may interfere with bacterial quorum sensing, thereby inhibiting biofilm formation. Its antioxidant properties neutralize free radicals, protecting host tissues from oxidative damage during infection.

**Key Mechanisms:**

**a.**  **Anti-Adhesive Properties:** The negatively charged sulfate groups in ulvan interact with positively charged surface molecules on bacterial cells, hindering their adhesion to host tissues or surfaces.
**b.**  **Biofilm Inhibition:** By preventing bacterial adhesion, ulvan suppresses biofilm formation and may destabilize existing MRSA biofilms.
**c.**  **Immune Modulation:** Ulvan enhances host immunity by activating macrophages and neutrophils, increasing phagocytosis and cytokine production to combat infections.

Antioxidant Activity: Ulvan scavenges free radicals, reducing oxidative stress and inflammation caused by MRSA.

**d.**  **Direct Antibacterial Effects:** Though secondary, ulvan may disrupt bacterial cell membranes, contributing to its antimicrobial action.
**e.**  **Quorum Sensing Interference:** Ulvan impedes bacterial communication, critical for biofilm development and virulence, thereby attenuating MRSA pathogenicity.

The after mentioned mechanisms highlight ulvan’s multifaceted potential in targeting MRSA through both direct and host-mediated pathways. Ulvan extracted from *Ulva lactuca* used to inhibit bacterial adhesion and biofilm formation in vitro assays. Ulvan is used to stimulate the immune system in animal models of infection [119].

#### 4.4.2. Carrageenan

Carrageenan is a family of linear sulfated polysaccharides composed of repeating galactose and 3,6-anhydrogalactose units, with varying degrees of sulfation. The primary types are kappa (κ), iota (ι), and lambda (λ) carrageenan, each with distinct sulfation patterns. *Kappaphycus alvarezii* and *Eucheuma denticulatum* (red algae) are amongst main algal sources for obtaining carrageenan.

**Mechanism of Action Against MRSA:** Carrageenan’s antibacterial activity is attributed to its sulfated nature. The negatively charged sulfate groups can interact with the positively charged bacterial cell surface, disrupting its integrity and interfering with adhesion [120]. Carrageenan can also inhibit biofilm formation by blocking the adhesion of MRSA cells to surfaces [121]. It appears that iota carrageenan shows increased effectiveness compared to other types of Carrageenan and it also blocks sortase enzymes. Some studies suggest that carrageenan can enhance the activity of antibiotics against MRSA by increasing bacterial membrane permeability [122].

#### 4.4.3. Porphyran

Porphyran is a sulfated polysaccharide found in red algae, particularly in the *Porphyra* genus (nori seaweed). It is a complex galactan, meaning it is primarily composed of galactose-based sugars. Its backbone consists of alternating α-(1→3)-linked galactose and β-(1→4)-linked 3,6-anhydrogalactose units and smaller amounts of other sugars, such as xylose and glucose. It also contains sulfate groups (SO^3−^) attached to the sugar residues. The degree and position of sulfation vary depending on the algal species and environmental conditions. Its molecular weight varies depending on the source and extraction method, typically ranging from 50 kDa to 500 kDa. Main algal sources for obtaining porphyrin are *Porphyra umbilicalis*, *Porphyra yezoensis*, and *Porphyra tenera*.

**Mechanism of Action Against MRSA:**

**a.**  **Inhibition of Bacterial Adhesion:** The sulfated nature of porphyran is believed to play a key role in its antibacterial activity. The negatively charged sulfate groups can interact with positively charged molecules on the surface of bacterial cells, preventing their adhesion to host tissues or surfaces.
**b.**  **Biofilm Inhibition:** By preventing initial adhesion, porphyran can inhibit the formation of biofilms by MRSA. It may also disrupt existing biofilms.
**c.**  **Immune Modulation:** Some studies suggest that sulfated polysaccharides like porphyran can stimulate the immune system, enhancing the host’s ability to fight off bacterial infections. They may activate macrophages and other immune cells.
**d.**  **Interference with Cell Wall Synthesis:** Though less common, there is a potential that porphyran could interfere with enzymes involved in bacterial cell wall synthesis.

Disruption of cell membrane: Porphyran structure may disrupt the cellular membranes of *Staphylococcus aureus*.

While the research is still growing, some relevant information exists. The antioxidant and antiproliferative capabilities of porphyran extracted from *Porphyra yezoensis* (red algae) has offered critical insights into its structural and functional characteristics [123]. Though the study does not directly address MRSA, the documented anti-adhesive properties of porphyran could hold relevance in mitigating bacterial infections, as such mechanisms often disrupt pathogen colonization—a potential indirect strategy against MRSA.

#### 4.4.4. Fucoidan

Fucoidan is a complex sulfated polysaccharide rich in fucose (a deoxy sugar). The structure varies depending on the algal species, but it typically contains a backbone of α-(1→3)-linked or α-(1→4)-linked L-fucopyranose residues, with sulfate groups attached at various positions. Fucoidan, a sulfated polysaccharide found primarily in brown algae (*Laminaria japonica*, *Undaria pinnatifida*), has been studied for its potential antithrombotic, anticancer, anticoagulant, and antiviral properties [124].

**Mechanism of Action Against MRSA:** Fucoidan exhibits multiple mechanisms against MRSA. In vitro studies have demonstrated fucoidan’s ability to inhibit MRSA biofilm formation [125]. It achieves this by preventing the initial attachment of bacterial cells to surfaces [126]. Fucoidan can also reduce the expression of virulence factors, such as toxins and adhesion molecules, contributing to reducing the severity of infections [127]. In addition, fucoidan can modulate the host immune response by stimulating the production of cytokines and enhancing phagocytosis of MRSA [128]. Fucoidan may interfere with bacterial efflux pumps to prevent antibiotic resistance [129].

Fucoidan from *L. japonica* shows antibacterial activity against *S. aureus* and *E. coli* by interacting with membrane proteins, causing cell membrane breakdown and cell death [130]. Fucoidan demonstrates effectiveness against MRSA, with MIC and MBC values ranging from 64 to 512 and 256 to 2048 μg/mL, respectively. When combined with antibiotics like ampicillin, it shows synergistic effects, reducing MIC and MBC. Fucoidan’s biocompatibility and low toxicity make it a promising nutraceutical supplement for infection control [131].

#### 4.4.5. Laminarin

Laminarin is a storage polysaccharide found in brown algae, particularly *Laminaria digitata* (Oarweed); *Laminaria hyperborea*; *Saccharina japonica* (Kombu); and other *Laminaria* species. It is a β-glucan, meaning it is composed of glucose units (D-glucose) linked together by β-glycosidic bonds, forming a linear chain of β-(1→3)-linked glucose with branching occurring at the β-(1→6) positions. Some laminarin molecules are terminated by mannitol. Its molecular weight varies depending on the algal species and extraction method, typically ranging from 2000 to 5000 Da.

**Mechanism of Action Against MRSA (Indirect, Through Immunomodulation):** While laminarin itself does not directly kill MRSA, it possesses significant immunomodulatory properties that can enhance the host’s immune response to the infection.

**Activation of Immune Cells:** Laminarin binds to pattern recognition receptors (PRRs) on immune cells, such as macrophages, neutrophils, and dendritic cells. The main PRR involved is Dectin-1, a receptor specific for β-glucans.

**Cytokine and Chemokine Production:** Binding to Dectin-1 triggers the activation of intracellular signaling pathways, leading to the production and release of cytokines (e.g., TNF-α, IL-1β, IL-6, IL-10) and chemokines (e.g., CXCL10, CCL2). These cytokines and chemokines play a crucial role in recruiting and activating other immune cells to the site of infection.

**Enhanced Phagocytosis:** Laminarin enhances the phagocytic activity of macrophages and neutrophils, increasing their ability to engulf and kill MRSA bacteria.

Increased ROS Production: Laminarin stimulates the production of reactive oxygen species (ROS) by immune cells, which are toxic to bacteria.

Increased Expression of Antimicrobial Peptides (AMPs): Laminarin can induce the expression of antimicrobial peptides (AMPs) by immune cells and epithelial cells. AMPs are small peptides that have direct antibacterial activity.

**Prebiotic Effect:** Laminarin can also act as a prebiotic, promoting the growth of beneficial gut bacteria. A healthy gut microbiome can help to outcompete pathogens like MRSA and modulate the immune response.

Although the cited studies do not directly investigate laminarin’s efficacy against MRSA, they highlight its immunomodulatory properties—a mechanism likely critical in addressing MRSA infections. For instance, Rioux et al. [131] explored the prebiotic effects of laminarin derived from Laminaria digitata, suggesting its role in modulating host immunity. Laminarin from Irish brown seaweeds, extracted using acidic solutions and ultrasound, effectively inhibits bacteria like *Salmonella typhimurium*, *Listeria monocytogenes*, *S. aureus*, and *E. coli* [98,132].

### 4.5. Amino Acids and Peptides

Algae are notable sources of proteins, with protein concentrations varying by type and season. Red seaweed contains 10–30% protein by dry weight (DW), brown seaweeds 5–15%, and green seaweeds 3–47%, with the highest levels typically found in winter. Peptides are short chains of amino acids linked by peptide bonds. They can be linear, cyclic, or branched and may contain unusual amino acids. Common examples include microcystins and cyanopeptolins. *Microcystis aeruginosa*, *Anabaena*, and *Nostoc* are common sources of cyclic peptides. Peptides from marine algae have gained attention for their health benefits, including antibacterial, antihypertensive, antioxidant, and antidiabetic properties [134].

**Mechanism of Action Against MRSA:** Algal peptides exert their antibacterial action by causing the following:

**a.**  **Membrane Disruption:** Lipopeptides can insert into bacterial membranes and create pores, leading to leakage and cell death.
**b.**  **Protein Synthesis Inhibition:** Some peptides target ribosomes and block protein synthesis.
**c.**  **Quorum Sensing Inhibition:** Some peptides can interfere with quorum sensing signals, thus reducing virulence factor production.

Cyanopeptolins are cyclic depsipeptides (containing both amino acids and hydroxy acids) produced by cyanobacteria, particularly *Microcystis aeruginosa* and *Planktothrix agardhii*. The structure varies depending on the species and strain.

**Mechanism of Action Against MRSA**: Some cyanopeptolins exhibit antibacterial activity by inhibiting bacterial proteases, which are essential for bacterial growth and virulence. Others can disrupt cell membranes, leading to increased permeability and cell death. The specific mechanism depends on the structure of the cyanopeptolin [135].

Microcystins are cyclic heptapeptides (containing seven amino acids) produced by cyanobacteria, particularly *Microcystis*, *Anabaena*, and *Oscillatoria*. The structure varies depending on the species and strain, but they all contain a unique amino acid called Adda (3-amino-9-methoxy-2,6,8-trimethyl-10-phenyldeca-4,6-dienoic acid).

**Mechanism of Action Against MRSA:** While primarily known for their hepatotoxicity (inhibition of protein phosphatases in eukaryotic cells), some microcystin variants have shown in vitro antibacterial activity. They may disrupt bacterial cell membranes or interfere with protein synthesis. The specific mechanism and potency vary depending on the structure of the microcystin [136]. Microcystins are highly toxic and require extreme caution. Any therapeutic use would need to carefully balance the antibacterial benefits against the risks of toxicity.

Antimicrobial peptides (AMPs) from marine environments differ structurally from terrestrial AMPs due to the high salt content of the ocean, enabling them to survive in ion-rich conditions [137]. Marine AMPs have unique modifications, such as bromination and chlorination, that enhance their structural stability and bioactivity [138]. These modifications have attracted pharmaceutical interest, with some marine peptides already FDA-approved for therapeutic use [139].

AMPs interact with MRSA bacterial cell membranes to form pores, causing leakage and bacterial death, a mechanism that reduces the risk of resistance development, unlike traditional antibiotics [96]. Enzymatic hydrolysis is a preferred method for extracting bioactive peptides from algae without leaving solvent residues, yielding peptides with higher biological activity than their parent proteins [45]. Crypteins, peptides with cryptic bioactivity, are also emerging as potential therapeutic agents [140].

Algal sources are rich in proteins suitable for obtaining bioactive peptides. For instance, *Tetraselmis suecica* has shown strong antibacterial activity against *E. coli*, MRSA, and *Bacillus cereus* [96]. Modifications to amino acid residues, such as lysine and alanine, enhance AMP effectiveness against pathogens without harming human cells [141]. The antimicrobial peptide SP-1 from *Spirulina platensis* effectively inhibits *S. aureus* and *E. coli* and is non-toxic to blood cells [133].

Microalgae-derived peptides, such as those from *Saccharina longicruris*, inhibit *S. aureus* growth at low concentrations [142]. Cyanobacteria also yield potent antimicrobial peptides, including Kawaguchipeptins and Norharmane-HCl, which effectively target various pathogens such as *S. aureus* and *E. coli* [143].

### 4.6. Lectins

Lectins are proteins or glycoproteins that bind specifically and reversibly to carbohydrates. Lectins are primarily proteins, meaning they are composed of amino acids linked together by peptide bonds. Some lectins are also glycoproteins, meaning they have carbohydrate chains attached to the protein backbone. Lectins contain specific carbohydrate-binding domains (CBDs) that recognize and bind to carbohydrate structures. Many lectins are multivalent, meaning they have multiple CBDs and can bind to multiple carbohydrate molecules simultaneously. This multivalent binding can lead to strong and specific interactions with cell surfaces. They are widespread in nature and are found in plants, animals, bacteria, and algae. They play important roles in cell recognition, adhesion, and signaling. Lectins have been identified in various algae species: *Griffithsia* sp. (red algae); *Ptilota plumosa* (red algae); *Codium tomentosum* (green algae); and *Bryopsis hypnoides* (green algae). In the context of anti-MRSA activity, the ability of lectins to bind to carbohydrates on bacterial cell surfaces is of primary interest.

**Mechanism of Action Against MRSA:** Lectins can potentially inhibit MRSA through several mechanisms:

**a.**  **Inhibition of Bacterial Adhesion:** A key initial step in MRSA infection is the adhesion of bacterial cells to host tissues. Lectins can bind to carbohydrates on the surface of MRSA cells, preventing them from adhering to host cells.
**b.**  **Agglutination of Bacterial Cells:** Because lectins are often multivalent, they can bind to multiple bacterial cells simultaneously, causing them to agglutinate (clump together). This agglutination can prevent MRSA from colonizing and spreading.
**c.**  **Interference with Biofilm Formation**: Biofilms are structured communities of bacteria encased in a matrix of extracellular polymeric substances (EPS). Lectins can interfere with biofilm formation by causing the following:
**d.**  **Preventing Initial Attachment:** As mentioned above, lectins can prevent MRSA cells from initially attaching to surfaces, a crucial step in biofilm formation.
**e.**  **Disrupting Biofilm Structure**: Lectins can bind to carbohydrates within the biofilm matrix, disrupting its structure and stability.
**f.**  **Immune Modulation:** Some lectins can activate the immune system, enhancing the body’s ability to fight off MRSA infections. They can stimulate the activity of macrophages, neutrophils, and other immune cells.
**g.**  **Direct Toxicity:** Though less common, some lectins may have direct toxic effects on bacterial cells, leading to cell death.
**h.**  **Disrupting cell wall synthesis:** Some lectins can bind to the cell wall of bacteria, therefore disrupting cell wall growth.

**Griffithsin** is a well-characterized lectin from red algae (*Griffithsia* sp.) that has been shown to have potent antiviral activity. While not specifically studied against MRSA, it serves as a model for the potential mechanisms of algal lectins. It binds to high-mannose oligosaccharides.

**Ptilota plumosa lectin**: This lectin has been shown to have anti-adhesive properties [144]. Unfortunately, the research directly linking algal lectins to specific anti-MRSA mechanisms is limited. Much of the research in this area focuses on lectins from other sources (e.g., plants) or examines the general antibacterial activity of algal extracts without isolating the specific lectin components.

### 4.7. Lipids and Fatty Acids

Lipids and fatty acids play an essential role in maintaining cellular structure and membrane integrity across many life forms. This makes them promising targets for developing novel anti-methicillin-resistant *Staphylococcus aureus* (MRSA) therapies. Lipid extracts from *Lyngbya* and *Acanthophora spicifera* also exhibit notable antibacterial activity against pathogens such as MRSA and *P. aeruginosa*, providing potential alternatives for antimicrobial treatments [145]. Fatty acids are long-chain carboxylic acids with varying degrees of saturation. Saturated fatty acids contain only single bonds between carbon atoms, while unsaturated fatty acids contain one or more double bonds. Polyunsaturated fatty acids (PUFAs) have multiple double bonds. Many algal species, including *Nannochloropsis* sp., *Phaeodactylum tricornutum*, and *Schizochytrium* sp., are rich in omega-3 fatty acids (Docosahexaenoic acid (DHA) and Eicosapentaenoic acid (EPA). Those derived from marine sources exhibit antimicrobial properties, being released from algal cells as a defense mechanism when cellular integrity is compromised, which can inhibit the growth of various infectious agents, including bacteria, viruses, and fungi [96]. Studies reveal that fatty acids possess bactericidal effects on various bacteria, with higher levels of carbon chain unsaturation enhancing these antibacterial properties [146]. Algal free fatty acids primarily target bacterial cell membranes, disrupting functions such as oxidative phosphorylation and the electron transport chain, thereby impeding bacterial growth and causing cellular destruction [147].

This review explores how specific algal fatty acids, such as docosahexaenoic acid (DHA), eicosapentaenoic acid (EPA), and palmitic acid, can combat MRSA through various mechanisms, including cell membrane disruption, interference with fatty acid metabolism, immune modulation, and inhibition of biofilm formation. Generally, polyunsaturated fatty acids (PUFAs) tend to be more effective than saturated fatty acids (SFAs) due to their greater impact on membrane fluidity [142].

**Mechanism of Action Against MRSA:** The antimicrobial effects of algal fatty acids against MRSA include the following:

**a.**  **Cell Membrane Disruption:** PUFAs can disrupt the bacterial cell membrane, increasing its fluidity and permeability, ultimately leading to cell death.
**b.**  **Interference with Fatty Acid Metabolism**: Fatty acids can disrupt the bacterial lipid metabolism pathways essential for cell structure and function.
**c.**  **Immune Modulation:** Certain fatty acids can modulate the host immune response, enhancing the body’s ability to fight off MRSA infection.

In Egypt, fatty acids from *Microcystis aeruginosa* demonstrate efficacy against *S. aureus*, *Klebsiella pneumoniae*, and *P. aeruginosa* [145]. Fatty acids from *Nostoc* and *Sargassum* species also show antibacterial effects, causing significant cell damage in resistant strains like *Klebsiella pneumoniae* and *S. aureus* [148]. Long- and short-chain fatty acids from *Scenedesmus obliquus* and *H. pluvialis*, respectively, exhibit antimicrobial properties, particularly against *S. aureus* and *E. coli* [149]. Additionally, *Stoechospermum marginatum* contains fatty acids and essential oils that exhibit antibacterial effects against drug-resistant bacteria such as *S. aureus* and *E. coli* [150].

#### 4.7.1. Docosahexaenoic Acid (DHA)

Docosahexaenoic acid (DHA) is an omega-3 polyunsaturated fatty acid with 22 carbons and 6 cis-double bonds, located at positions 4, 7, 10, 13, 16, and 19 from the methyl endCH_3_(CH_2_)_5_CH=CH(CH_2_)CH=CH(CH_2_)CH=CH(CH_2_)CH=CH(CH_2_)CH=CH(CH_2_)_3_COOH). It is a carboxylic acid with a long aliphatic chain. *Crypthecodinium cohnii* and *Schizochytrium* sp. are both marine protists, often classified as algae in a broader sense, particularly for industrial production of omega-3 fatty acids.

**Mechanism of Action Against MRSA**: Like EPA, DHA’s primary mechanism is disrupting the MRSA cell membrane. The incorporation of DHA into the bacterial phospholipid bilayer alters its fluidity and permeability. This increased membrane permeability leads to leakage of essential cellular components, disrupting ion gradients, and impairing the function of membrane-bound proteins. DHA can also interfere with quorum sensing (QS) in MRSA, reducing the production of virulence factors such as toxins and biofilm formation [151]. Furthermore, studies suggest that DHA can modulate the host immune response, promoting phagocytosis of MRSA by macrophages and reducing inflammation caused by MRSA infection [152]. Docosahexaenoic acid (DHA) could help prevent MRSA biofilm formation, with *Fischerella* sp.-derived γ-linolenic acid (GLA) proving effective against *S. aureus* [153].

**Limitations and Considerations:** DHA is susceptible to oxidation, potentially leading to a loss of activity. Encapsulation may be necessary to improve stability and delivery. There are also bioavailability concerns, including how well it is absorbed and reaches the infection site [154].

#### 4.7.2. Eicosapentaenoic Acid (EPA)

Eicosapentaenoic acid (EPA) is an omega-3 polyunsaturated fatty acid with 20 carbons and 5 cis-double bonds, located at positions 5, 8, 11, 14, and 17 from the methyl end (CH_3_(CH_2_)_3_(CH_2_CH=CH)_5_ (CH_2_)_3_COOH). *Phaeodactylum tricornutum* (diatom) and *Nannochloropsis* sp. (eustigmatophyte) are rich in EPA.

**Mechanism of Action Against MRSA**: EPA integrates into the MRSA cell membrane, increasing fluidity and permeability, which can disrupt membrane function and lead to cell death. EPA also exhibits anti-biofilm activity against MRSA by inhibiting the expression of genes involved in biofilm formation, such as icaA (intercellular adhesion gene A) and icaD (intercellular adhesion gene D) (encoding enzymes involved in polysaccharide intercellular adhesin (PIA) synthesis, a major component of MRSA biofilms). EPA has shown the ability to inhibit the fatty acid biosynthesis pathway in *Staphylococcus aureus*, inhibiting cell wall growth [154]. EPA effectively inhibits the growth of *S. aureus* and *Bacillus cereus*, likely causing bacterial membrane disruption and cell lysis at higher concentrations [155].

**Limitations and Considerations**: Like DHA, EPA faces challenges related to stability and bioavailability. However, EPA and DHA have demonstrated synergistic effects against MRSA. The combination of EPA and DHA has been shown to increase the sensitivity of MRSA to other antibiotics [156].

#### 4.7.3. Palmitic Acid

Palmitic acid is a saturated fatty acid with 16 carbons (CH_3_(CH_2_)_14_COOH). It is a straight-chain carboxylic acid. *Chlorella vulgaris* (green algae) and *Spirulina* sp. (*Arthrospira platensis*; cyanobacteria) are the main algal sources for obtaining palmitic acid.

**Mechanism of Action Against MRSA:** Palmitic acid’s antibacterial activity against MRSA likely involves disruption of the cell membrane, although it is generally less potent than unsaturated fatty acids. The insertion of palmitic acid into the phospholipid bilayer can increase membrane permeability, leading to leakage of cellular contents. It can also interfere with membrane-bound enzymes and transport proteins. Palmitic acid is thought to inhibit ATP production as well as various other enzymes necessary for the metabolic pathways of *Staphylococcus aureus* [157].

**Limitations and Considerations:** Palmitic acid is less effective than unsaturated fatty acids like DHA and EPA against MRSA. However, it has demonstrated synergistic effects with unsaturated fatty acids, increasing the overall effectiveness of algal extracts. Some studies show conflicting results regarding the antibacterial activity of palmitic acid or show no effect at all [157].

#### 4.7.4. Other Fatty Acids (e.g., Hexadecatrienoic Acid, Palmitoleic Acid)

Fatty acids such as hexadecatrienoic acid (HTA), eicosapentaenoic acid (EPA), and palmitoleic acid (PA) from *Phaeodactylum tricornutum* demonstrate strong antimicrobial effects against Gram-positive and Gram-negative bacteria, including MRSA, *Staphylococcus aureus*, and *Vibrio* species, with EPA proving effective even at low concentrations [158]. Further studies highlight that omega-3 fatty acids like EPA and DHA significantly reduce *S. aureus* biofilm formation and virulence, with transcriptional analysis indicating suppression of the hla gene, which encodes α-hemolysin [189]. These findings suggest that algal fatty acids attack various sites in bacterial cells, making them less susceptible to resistance and offering potential as alternative treatments in drug discovery [96].

### 4.8. Glycolipids

Glycolipids are lipids with one or more covalently attached carbohydrate molecules. The lipid portion of a glycolipid can be a glycerol-based lipid (e.g., diacylglycerol) or a sphingoid base (e.g., ceramide), while the carbohydrate portion can consist of one or more monosaccharide units (e.g., glucose, galactose, mannose) linked together to form oligosaccharides. The carbohydrate is attached to the lipid moiety via a glycosidic bond. There is a wide variety of glycolipid structures depending on the type of lipid, the type and number of sugars, and the linkages between them. Glycolipids are found in various algae species: *Chlorella* sp. (green algae); *Spirulina* sp. (cyanobacteria but often grouped with algae in this context); *Porphyridium cruentum* (red algae); *Ochromonas danica* (golden algae); and *Euglena gracilis* (euglenoid). They are important components of cell membranes, where they play roles in cell recognition, cell signaling, and membrane stability. Glycolipids have both a hydrophobic (lipid) portion and a hydrophilic (carbohydrate) portion, giving them an amphiphilic nature. This allows them to insert into cell membranes and interact with both the lipid and aqueous environments. In the context of anti-MRSA activity, the amphiphilic nature of glycolipids and their ability to interact with cell membranes are of primary interest.

#### 4.8.1. Sulfoquinovosyl Diacylglycerol (SQDG)

Sulfoquinovosyl Diacylglycerol (SQDG): A sulfolipid found in photosynthetic organisms including cyanobacteria and algae. It disrupts membrane function and has shown general antibacterial activity in several species [190].

#### 4.8.2. Monogalactosyl Diacylglycerol (MGDG)

Monogalactosyl Diacylglycerol (MGDG): Found in the thylakoid membranes of plants, algae, and cyanobacteria. It exhibits broad antimicrobial potential by disrupting cellular function [191].

#### 4.8.3. Digalactosyl Diacylglycerol (DGDG)

Digalactosyl Diacylglycerol (DGDG): Found in plants, algae, and cyanobacteria and has exhibited potential anti-inflammatory activity [192].

**Mechanism of Action Against MRSA**: Glycolipids can potentially inhibit MRSA through several mechanisms:

**a.**  **Disruption of Cell Membrane Integrity:** The amphiphilic nature of glycolipids allows them to insert into the bacterial cell membrane.
**b.**  **Increase Membrane Permeability:** Glycolipids can increase the permeability of the cell membrane, leading to leakage of essential cellular components and disruption of ion gradients.
**c.**  **Alter Membrane Fluidity:** Glycolipids can alter the fluidity of the cell membrane, affecting the function of membrane proteins and transport systems.
**d.**  **Inhibition of Bacterial Enzymes:** Some glycolipids can inhibit bacterial enzymes involved in cell wall synthesis or other essential metabolic pathways.
**e.**  **Interference with Biofilm Formation**: Glycolipids can interfere with biofilm formation by preventing initial attachment as glycolipids can coat surfaces and prevent MRSA cells from initially attaching.
**f.**  **Disrupting Biofilm Structure:** Glycolipids can insert into the biofilm matrix, disrupting its structure and stability.
**g.**  **Modulation of Host Immune Response:** Some glycolipids may have immunomodulatory properties, enhancing the host’s ability to clear MRSA infections. They may stimulate the activity of immune cells such as macrophages.

**Inhibition of Sortase Enzymes:** Sortase enzymes are responsible for anchoring surface proteins, which can contribute to the pathogenesis of infections.

Unfortunately, as with some other algal metabolites, there is a relative lack of specific research directly linking algal glycolipids to specific anti-MRSA mechanisms. Much of the research tends to focus on the general antibacterial activity of algal extracts or on glycolipids from other sources [193].

### 4.9. Terpenoids

Terpenoids (also known as isoprenoids) constitute an incredibly diverse class of natural products found in different species of algae such as: *Sargassum* sp., *Dictyota dichotoma*, *Dictyopteris* sp., *Fucus* sp. (brown algae); *Ulva lactuca* (green algae); and *Laurencia* sp. (red algae). Terpenoids are a diverse class of natural products derived from isoprene (C_5_H_8_) units. They can be linear or cyclic and can be further modified by oxidation, hydroxylation, and other chemical reactions. Terpenoids’ classification based on isoprene units into monoterpenes (C_10_H_16_) which consist of two isoprene units; sesquiterpenes (C_15_H_24_) which consist of three isoprene units; diterpenes (C_20_H_32_) which consist of four isoprene units; sesterterpenes (C_25_H_40_) which consist of five isoprene units; triterpenes (C_30_H_48_) which consist of six isoprene units; and tetraterpenes (C_40_H_64_) which consist of eight isoprene units. They exhibit a wide range of biological activities.

**Mechanism of Action Against MRSA:** The mechanism of action varies significantly depending on the specific terpenoid structure.

Common mechanisms include the following:

**a.**  **Disruption of Cell Membrane Integrity:** Many terpenoids are lipophilic and can insert themselves into the bacterial cell membrane, altering its fluidity, permeability, and function. This can lead to leakage of cellular contents, disruption of ion gradients, and, ultimately, cell death.
**b.**  **Inhibition of Bacterial Enzymes:** Some terpenoids can inhibit key bacterial enzymes involved in essential metabolic pathways or cell wall synthesis. For example, some terpenoids can inhibit peptidoglycan synthesis, a crucial step in bacterial cell wall formation.

Interference with Quorum Sensing (QS): QS is a cell-to-cell communication system used by bacteria to coordinate gene expression, biofilm formation, and virulence factor production. Certain terpenoids can interfere with QS signaling pathways, reducing biofilm formation and virulence.

**c.**  **Efflux Pump Inhibition:** Some terpenoids can inhibit bacterial efflux pumps, which are membrane proteins that pump antibiotics out of the bacterial cell. By inhibiting efflux pumps, terpenoids can increase the intracellular concentration of antibiotics, making MRSA more susceptible to these drugs.
**d.**  **Protein Synthesis Inhibition:** Terpenoids can interfere with bacterial protein synthesis, disrupting the production of essential proteins required for bacterial growth and survival.

DNA Replication Interference: Some terpenoids can interfere with bacterial DNA replication, preventing the bacteria from multiplying.

#### 4.9.1. Sargachromanol E

Sargachromanol E: A prenylated chromanol derivative with a molecular formula of C_26_H_38_O_4_. It contains a chromanol ring system with a prenyl group attached. Sargachromanol E from *Sargassum yezoense* has potent antibacterial activity against MRSA. It disrupts the bacterial membrane, leading to increased permeability and cell death. It can also inhibit the synthesis of essential bacterial proteins and interfere with bacterial efflux pumps, increasing the intracellular concentration of antibiotics. This latter effect can reverse antibiotic resistance in MRSA [159].

#### 4.9.2. Dictyopterene A

Brown algae from the genus *Dictyopteris* are known to produce a variety of volatile compounds, including cyclic olefins like dictyopterenes. While these compounds are primarily studied for their role as pheromones, crude extracts from species such as *Dictyopteris divaricata* have demonstrated significant antibacterial properties. In a screening of various Korean marine algae, methanolic extracts from *D. divaricata* exhibited the most potent inhibitory activity against methicillin-resistant *Staphylococcus aureus* (MRSA) strains, suggesting that this alga is a promising source of anti-MRSA compounds warranting further investigation [160].

#### 4.9.3. Caulerprenyne

Caulerprenyne: Acyclic diterpene from *Caulerpa taxifolia* possesses the potential to disrupt cell membrane of Gram-positive bacteria (anti-microbial) [161].

#### 4.9.4. Sargaquinoic Acid

Sargaquinoic acid from the brown alga *Sargassum micracanthum* was found to exhibit strong antimicrobial activity against MRSA strains by inhibiting DNA polymerase α [11].

Algal diterpene-benzoates, such as bromophycolides from *Callophycus serratus*, inhibit bacterial growth, demonstrating activity against MRSA and vancomycin-resistant *Enterococcus faecium* with IC50 values of 1.4 μM and 5.8 μM, respectively [162]. Brominated diterpenes like sphaerodactylomelol and sphaerane derived from *Sphaerococcus coronopifolius* showed strong activity, particularly against *Staphylococcus aureus*, with an IC50 of 6.35 μM [86].

*Staphylococcus aureus* growth was inhibited by terpenes from *Sphaerococcus coronopifolius*, including 12S-hydroxybromosphaerodiol and bromosphaerone, with MICs of 0.104 μg/mL and 0.146 μg/mL, respectively. The amphipathic structure of bromosphaerone enables it to interact with cell membranes, enhancing its antibacterial effect [163].

Additionally, the sesquiterpenoid neophytadiene, found in *Ulva lactuca*, displayed antimicrobial efficacy against multidrug-resistant *Staphylococcus aureus*, *Klebsiella pneumoniae*, and *Escherichia coli*, highlighting terpenes’ potential for diverse therapeutic applications [150]. These varied properties underscore the terminus’ utility in developing new antimicrobial treatments [96].

### 4.10. Saponins

Saponins are glycosides with a characteristic soap-like foaming ability when mixed with water. Saponins are glycosides, meaning they consist of hydrophilic sugar (glycone) molecule(s) bound to hydrophobic non-sugar (aglycone) molecules. The sugar chains (glycone) are typically composed of monosaccharides such as glucose, galactose, xylose, arabinose, rhamnose, or glucuronic acid. These sugars can be attached to the aglycone at one or more positions. The aglycone (Sapogenin) can be either a triterpenoid (C30) or a steroid (C27) structure. Triterpenoid saponins are more common in plants, but algal saponins can be either triterpenoid or steroid-based. They are found in a variety of plants, and while less common in algae than some other compound classes, they have been identified in some algal species like *Enteromorpha compressa* (green algae); *Chaetomorpha linum* (green algae); and *Sargassum wightii* (brown algae). The specific saponin content and type can vary greatly depending on the algal species, the geographic location, and the environmental conditions.

The combination of the hydrophobic aglycone and the hydrophilic sugar chains give saponins their amphipathic nature, allowing them to interact with both water and lipids. This amphipathic property is responsible for their foaming ability and their ability to disrupt cell membranes.

**Mechanism of action against MRSA:**

**a.**  **Disruption of Cell Membranes:** The primary mechanism of action of saponins against bacteria, including MRSA, is the disruption of cell membranes. The amphipathic nature of saponins allows them to insert themselves into the lipid bilayer of bacterial cell membranes.
**b.**  **Increase Membrane Permeability**: Saponins can increase the permeability of the cell membrane, leading to leakage of essential cellular components (e.g., ions, proteins, nucleotides).
**c.**  **Cause Membrane Disruption and Lysis:** At higher concentrations, saponins can cause complete disruption and lysis (bursting) of the cell membrane.
**d.**  **Interaction with Membrane Proteins:** Saponins can also interact with membrane proteins, disrupting their function and leading to cell death.
**e.**  **Inhibition of Bacterial Enzymes:** Some saponins can inhibit bacterial enzymes involved in essential metabolic pathways, such as cell wall synthesis or DNA replication. However, this mechanism is less specifically studied for algal saponins specifically.
**f.**  **Biofilm Inhibition:** Several saponins have been shown to have anti-biofilm activity, preventing the formation of biofilms by bacteria. This can be particularly important for MRSA, which often forms biofilms that protect it from antibiotics and the host’s immune system.
**g.**  **Immunomodulatory Effects:** Some saponins can stimulate the immune system, enhancing the body’s ability to fight off bacterial infections. However, this mechanism is more relevant to in vivo (animal or human) studies.

Due to the relative lack of research on algal saponins compared to plant saponins, identifying specific examples with well-defined anti-MRSA mechanisms is challenging. To extrapolate and build a more detailed potential anti-MRSA effect, one could analyze saponins derived from similar ecological environments to algae, sea cucumbers for example. Holothurin A: While technically from sea cucumbers (marine animals), holothurins are saponins that have been extensively studied for their antibacterial and anticancer properties. Holothurin A has displayed a great capacity to act against Gram-positive bacteria, which include *Staphylococcus aureus*. This is achieved by disruption of cellular membrane and inhibition of biofilm formation [164].

The specific mechanisms of action of algal saponins against MRSA need to be further investigated. Exploring the synergistic effects of algal saponins with existing antibiotics could be a promising approach. The bioavailability and toxicity of algal saponins need to be assessed. Saponins can be toxic at high concentrations, so careful evaluation is necessary.

In summary, algal saponins are a potentially valuable source of antibacterial compounds, but more research is needed to fully understand their potential against MRSA. Because the research on algal saponins and their direct anti-MRSA activity is quite limited, some of the information above is extrapolated from studies on saponins from other sources (like plants or sea cucumbers). Further research is needed to confirm these mechanisms for algal saponins specifically.

### 4.11. Sterols (Fucosterol)

Fucosterol from *Fucus vesiculosus* (brown algae) is technically a sterol with a unique side chain but derived from the terpenoid pathway. It disrupts the bacterial cell membrane and inhibits biofilm formation [93]. Its chemical formula is C_29_H_48_O. It is structurally like cholesterol but contains a unique ethylidene group at position C-24.

**Mechanism of Action Against MRSA:** Fucosterol’s antibacterial activity is thought to involve disruption of the bacterial cell membrane, leading to increased permeability and cell death. It can also inhibit biofilm formation by interfering with bacterial adhesion and quorum sensing. Some studies suggest that fucosterol can enhance the activity of antibiotics against MRSA by increasing bacterial membrane permeability [93]. Fucosterol decreases the cellular metabolic activity that is necessary for *Staphylococcus aureus* growth and biofilm formation.

### 4.12. Chrysophaentins

**Figure 11:**
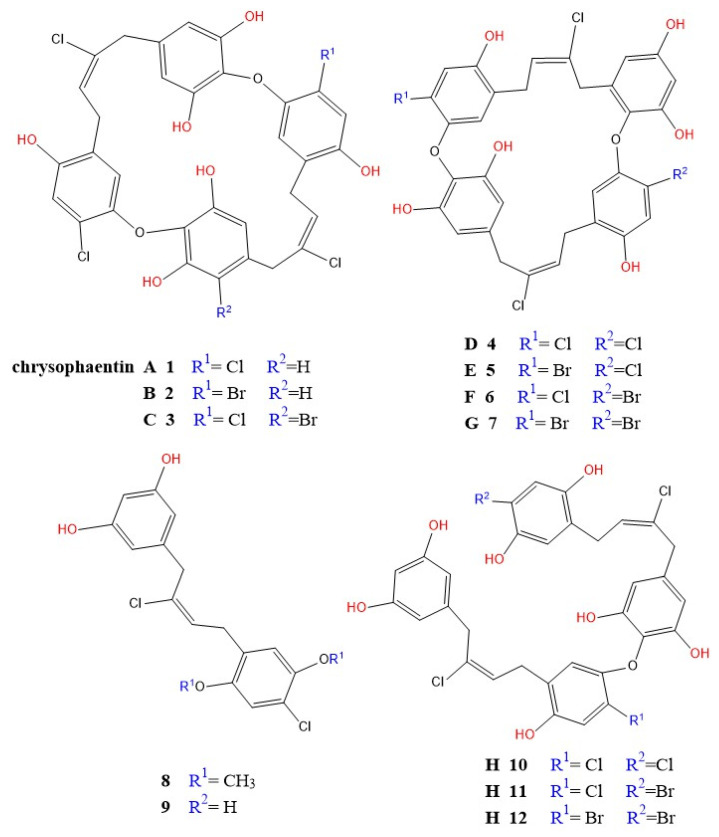
The structures of synthesized components and biological chrysophaentins.

Chrysophaentins are a class of eight new antibacterial compounds, chrysophaentins A-H, isolated from the marine alga *Chrysophaeum taylori* through hexane, chloroform, and methanol extraction methods by Plaza et al. [165]. These compounds are characterized by a unique chemical structure, comprising two polyhydroxylated, polyhalogenated ω,ω1-diarylbutene units connected by two ether linkages, distinguishing them within antibacterial agents (see Figure 11).

Chrysophaentins operate by a novel antibacterial mechanism, different from traditional drugs. They act as enzyme inhibitors by binding to bacterial GTPase, disrupting the function of FtsZ, a protein critical for bacterial cell division. This inhibition stops the formation of the Z ring, essential for initiating bacterial cell division [96]. Through biochemical assays and molecular docking, chrysophaentins were found to competitively inhibit GTP binding to FtsZ, preventing bacterial cell division and thus demonstrating potent antibacterial action [167].

The antibacterial efficacy of chrysophaentins was evident in vitro, as they inhibited MRSA at a minimum inhibitory concentration (MIC) of 1.5 μg/mL, vancomycin-resistant *Enterococcus faecium* at an MIC of 2.9 μg/mL, and multidrug-resistant *Staphylococcus aureus* at an MIC of 1.3 μg/mL [194]. Keffer et al. highlighted the broad-spectrum activity of chrysophaentins, showing efficacy against various *S. aureus* strains, including methicillin-resistant strains, due to their bisdiarylbutene macrocycle structure [166].

The novel action of chrysophaentins underscores their potential in developing antibiotics to address global drug resistance, representing a significant advancement in combating resistant bacterial strains [167].

### 4.13. Other Metabolites

#### Acrylic Acid

Acrylic acid (also known as propenoic acid) is a simple unsaturated carboxylic acid with molecular formula of C_3_H_4_O_2_ and chemical structure of CH_2_=CHCOOH. It is naturally produced by some algae [194]. *Phaeocystis pouchetii* (Prymnesiophyte/Haptophyte) is one of the best-known algal producers of acrylic acid [195]. *Emiliania huxleyi* (Prymnesiophyte/Haptophyte) is another important phytoplankton species that produces acrylic acid [196]. The production of acrylic acid often increases during algal blooms. The antibacterial properties of acrylic acid have been recognized for a long time [197].

**Mechanism of Action Against MRSA:**

**Disruption of Bacterial Metabolism**: Acrylic acid is a broad-spectrum antimicrobial agent that disrupts bacterial metabolism. It can inhibit bacterial enzymes involved in essential metabolic pathways [198].

**Inhibition of Protein Synthesis**: Acrylic acid can interfere with protein synthesis in bacteria, leading to growth inhibition and cell death [198].

**Cell Membrane Disruption:** At higher concentrations, acrylic acid can disrupt the bacterial cell membrane, increasing permeability and causing leakage of cellular components [199].

**pH Reduction:** As an acid, acrylic acid can lower the pH of the bacterial environment, which can inhibit bacterial growth [200].

**Inhibition of Amino Acid Transport:** A more targeted study shows that acrylate inhibits the bacterial enzyme alanine racemase in *Staphylococcus aureus*, preventing the growth and cell wall assembly [201].

## 5. Recent Advances, Challenges, and Future Directions

The field of marine-derived anti-MRSA agents is advancing rapidly, with a strategic shift towards developing more sophisticated therapies. This section will first highlight key trends from the recent literature before critically analyzing the major hurdles that must be overcome for clinical translation.

### 5.1. Emerging Therapeutic Strategies

Research published in 2024 and 2025 illustrates a clear focus on innovative therapeutic strategies. A significant trend is the targeting of specific virulence factors to disarm the pathogen; for example, a novel diterpenoid isolated from the red alga *Laurencia L.* was shown to be a potent inhibitor of the MRSA sortase A (SrtA) enzyme, which is crucial for anchoring surface proteins involved in host tissue adhesion [202]. Another major strategy is the use of synergistic combinations to rejuvenate existing antibiotics. A study demonstrated that a low-molecular-weight sulfated polysaccharide from the green alga *Ulva lactuca* significantly disrupted the MRSA biofilm matrix, thereby potentiating the effect of linezolid and lowering its effective MIC four-fold [203]. The exploration of underexplored marine microorganisms also continues to yield novel chemical scaffolds, such as the chlorinated alkaloids discovered in the dinoflagellate *Amphidinium carterae*, which showed strong activity against vancomycin-intermediate *S. aureus* (VISA) strains [204]. Furthermore, the integration of nanotechnology is creating advanced therapeutic platforms. Fucoidan-functionalized selenium nanoparticles (Fu-SeNPs) were developed and shown to not only exhibit direct antibacterial effects but also to mitigate the inflammatory response in a murine MRSA-infected wound model, demonstrating a promising dual-action therapeutic approach [205].

### 5.2. Key Developmental Hurdles

While the therapeutic potential of algal metabolites is significant, their transition from laboratory discovery to clinical application is fraught with challenges. A critical analysis of these hurdles is essential for guiding future research.

#### 5.2.1. Bioavailability and Drug Delivery

A major hurdle for many promising algal metabolites, particularly lipophilic compounds like certain terpenes and polyphenols, is their poor bioavailability. Low water solubility can impede absorption, while rapid metabolism in the liver can prevent the compound from reaching therapeutic concentrations at the site of infection. As highlighted in recent 2024 studies on marine drug delivery, future research must focus on developing advanced systems, such as nano-formulations (e.g., liposomes, polymeric nanoparticles), to enhance the solubility, stability, and targeted delivery of these potent agents [206].

#### 5.2.2. Potential Toxicity

While targeting bacterial cells, some potent algal compounds can exhibit off-target cytotoxicity against mammalian cells, limiting their therapeutic window. For instance, certain alkaloids and peptides like microcystins are known for their toxicity, a point of concern that continues to be a focus in toxicological studies into 2025 [207]. Therefore, rigorous preclinical safety and toxicology profiling are essential to determine the therapeutic index, establish safe dosage levels, and identify any potential adverse effects before these compounds can be considered for human trials.

#### 5.2.3. Scalability and Sustainable Production

The translation from a laboratory discovery to a commercial drug requires a large-scale, sustainable, and cost-effective supply of the active compound. Many bioactive metabolites are found in minute quantities in algae, making extraction from wild harvests unsustainable and purification challenging and expensive. Recent advances in algal biotechnology, including optimizing cultivation conditions in photobioreactors and applying metabolic engineering, are crucial to overcoming these scalability challenges and ensuring a consistent supply chain for future drug development, a key theme in sustainable biotechnology research in 2024 [208].

#### 5.2.4. Clinical Validation

A significant gap in the current research is the translation of promising in vitro results to in vivo efficacy. Many compounds that effectively kill MRSA in a test tube fail in animal models due to poor pharmacokinetics, toxicity, or instability. There is a critical need for more robust preclinical studies in relevant animal infection models, such as the MRSA-infected wound models often used in 2024 and 2025 studies, to validate the therapeutic potential and safety of these algal metabolites before they can advance to clinical development [209]. This gap between lab findings and clinical reality remains the “valley of death” for many natural products.

## 6. Conclusions

This review underscores the escalating global health crisis posed by methicillin-resistant *Staphylococcus aureus* (MRSA), a formidable “superbug” armed with a diverse arsenal of resistance mechanisms. We have detailed the prevalent traits of *S. aureus*, the concerning emergence and spread of MRSA, the modes of action of conventional antibiotics, and the multifaceted strategies MRSA employs to evade these treatments. The limitations of current therapeutic options, even last-resort drugs like vancomycin, highlight the critical and urgent need for novel antimicrobial agents.

Marine algae, a largely untapped reservoir of chemical diversity, presents a compelling avenue for the discovery of such novel therapeutics. This review has systematically explored a wide array of algal metabolites, including polyphenols, alkaloids, pigments, polysaccharides, peptides, lectins, lipids, terpenoids, and unique compounds like chrysophaentins. These metabolites exhibit potent anti-MRSA activity through diverse and often multi-target mechanisms, such as disrupting cell membranes, inhibiting protein synthesis, interfering with biofilm formation and quorum sensing, and targeting novel sites like the FtsZ protein involved in cell division.

The ability of these algal-derived compounds to counteract multiple resistance mechanisms, coupled with advances in algal biotechnology for sustainable production, positions marine algae as a highly promising source for the development of innovative therapeutic strategies. Future research must focus on in-depth mechanistic studies, in vivo efficacy and safety evaluations, and the development of optimized extraction and delivery systems. For instance, innovative strategies are already being explored, as exemplified by a 2024 study where algae-mediated silver nanoparticles were used to create a potent anti-biofilm platform against clinical MRSA isolates [210]. Translating the considerable potential of these compounds through such advanced approaches will be crucial in the ongoing battle against antibiotic resistance and the quest for new weapons to combat resilient pathogens like MRSA.

## Figures and Tables

**Table 1:** Comprehensive summary of algal metabolites with potential anti-MRSA activity.

Chemical Group	Specific Compound/Sub-Class	Algal Source(s)	Mechanism of Action Against MRSA	Key Findings and MIC Values	Developmental Considerations	Reference(s)
Polyphenols	Phlorotannins (e.g., Eckol)	Brown algae (*Padina australis*, *Ecklonia kurome*, *Eisenia bicyclis*)	Cell membrane disruption; inhibition of methicillin-resistance genes; biofilm and quorum sensing (QS) interference; enzyme and nucleic acid inhibition.	MIC values as low as 32–64 µg/mL against MRSA (*E. bicyclis*).	Efficacy is influenced by the degree of polymerization.	[80,81,82,83,84,85,86,87,88,89]
Polyphenols	Bromophenols	Red algae (*Rhodomela larix*, *Kappaphycus* sp.)	Cell membrane disruption; quorum sensing (QS) inhibition; protein inhibition.	Effective against MRSA and other *Staphylococcus* species.	Widespread across macroalgal taxa with diverse structures.	[90,91,92,93,94,95]
Alkaloids	Cyanobacterial Alkaloids (Hapalindoles, N-methylcytisin)	Cyanobacteria (*Hapalosiphon* sp., *Fischerella* sp., *Nostoc* sp.)	QS interference; efflux pump suppression; DNA intercalation/polymerase suppression.	N-methylcytisin showed potent action against *S. aureus* at 150 µg/mL.	Research on specific anti-MRSA activity is less extensive than other classes.	[96,97,98,99,100]
Alkaloids	Caulerpin	Green algae (*Caulerpa taxifolia*)	Disruption of cell membrane function; inhibition of cell division.	Strong antibacterial effects reported, particularly against resistant strains like MRSA.	A bisindole alkaloid with a lipophilic nature allowing membrane insertion.	[101,102]
Pigments	Carotenoids, Chlorophylls, Phycobiliproteins	Green, red, brown algae and cyanobacteria (*Spirulina*, *Haematococcus*, etc.)	Direct action (membrane disruption, cell wall interference) and indirect action (ROS generation, immunomodulation, biofilm inhibition).	Fucoxanthin is effective against *S. aureus*; Astaxanthin inhibits biofilm.	Many act as antioxidants, a host-mediated benefit, rather than being directly bactericidal.	[96,103,104,105,106,107,108,109,110,111,112,113,114,115,116,117]
Polysaccharides (Sulfated)	Fucoidan, Laminarin, Ulvan, Carrageenan	Brown algae (*Laminaria*), green algae (*Ulva*), red algae (*Kappaphycus*)	Anti-adhesion and biofilm disruption; immune modulation (activating macrophages); efflux pump interference (Fucoidan).	Fucoidan shows synergistic effects with ampicillin; MIC against MRSA ranges from 64 to 512 µg/mL.	Activity depends on molecular weight and degree of sulfation; Some have primarily indirect effects.	[98,118,119,120,121,122,123,124,125,126,127,128,129,130,131,132]
Peptides and Amino Acids	Antimicrobial Peptides (AMPs), Cyanopeptolins	Cyanobacteria (*Microcystis*), green algae (*Tetraselmis*)	Direct membrane disruption via pore formation; inhibition of protein synthesis or proteases; QS inhibition.	SP-1 peptide from *Spirulina* is non-toxic to blood cells.	High toxicity is a major concern for some compounds like microcystins.	[96,98,133,134,135,136,137,138,139,140,141,142,143]
Lectins	Griffithsin, Ptilota plumosa lectin	Red algae (*Griffithsia* sp.), green algae (*Codium*)	Inhibition of bacterial adhesion; agglutination of bacterial cells; biofilm formation interference.	Research on *algal* lectins specifically against MRSA is limited; mechanisms are often extrapolated.	-	[144]
Lipids and Fatty Acids	PUFAs (DHA, EPA) and SFAs (Palmitic acid)	Diatoms (*Phaeodactylum*), green Algae (*Chlorella vulgaris*)	Cell membrane disruption by altering fluidity; interference with fatty acid metabolism; biofilm and QS inhibition.	PUFAs are generally more potent than SFAs but face challenges with oxidation and bioavailability.	-	[96,145,146,147,148,149,150,151,152,153,154,155,156,157,158]
Terpenoids	Sargachromanol E, Bromophycolides	Brown algae (*Sargassum*), red algae (*Callophycus*)	Disruption of cell membrane; inhibition of DNA polymerase and other enzymes; efflux pump inhibition.	Bromophycolides show potent activity with IC50 values in the low µM range against MRSA.	An incredibly diverse class with a wide range of activities depending on structure.	[11,84,96,150,159,160,161,162,163]
Saponins	(e.g., Holothurins used as model)	Green andbrown algae (*Enteromorpha*, *Sargassum*)	Primary: strong cell membrane disruption leading to lysis; biofilm inhibition.	Research on *algal* saponins is very limited; can be toxic at high concentrations.	-	[164]
Sterols	Fucosterol	Brown algae (*Fucus vesiculosus*)	Disrupts bacterial cell membrane; inhibits biofilm formation; decreases cellular metabolic activity.	Can enhance the activity of conventional antibiotics.	-	[93]
Chrysophaentins	Chrysophaentins A-H	Marine alga *Chrysophaeum taylori*	Novel Mechanism: inhibits the FtsZ protein (a bacterial GTPase), preventing Z-ring formation and halting cell division.	Potent activity with MIC of 1.5 µg/mL against MRSA.	Represents a significant advancement with a novel target, reducing the chance of cross-resistance.	[96,165,166,167]
